# The hump-shaped effect of plant functional diversity on the biological control of a multi-species pest community

**DOI:** 10.1038/s41598-021-01160-2

**Published:** 2021-11-04

**Authors:** Antoine Gardarin, Justine Pigot, Muriel Valantin-Morison

**Affiliations:** UMR Agronomie, INRAE, AgroParisTech, Université Paris-Saclay, 78 850 Thiverval-Grignon, France

**Keywords:** Agroecology, Biodiversity, Community ecology

## Abstract

Plant taxonomic and functional diversity promotes interactions at higher trophic levels, but the contribution of functional diversity effects to multitrophic interactions and ecosystem functioning remains unclear. We investigated this relationship in a factorial field experiment comparing the effect of contrasting plant communities on parasitism rates in five herbivore species. We used a mechanistic trait-matching approach between plant and parasitoids to determine the amount of nectar available and accessible to parasitoids. This trait-matching approach best explained the rates of parasitism of each herbivorous species, confirming the predominant role of mass-ratio effects. We found evidence for an effect of functional diversity only in analyses considering the ability of plant communities to support the parasitism of all herbivores simultaneously. Multi-species parasitism was maximal at intermediate levels of functional diversity. Plant specific richness had a negligible influence relative to functional metrics. Plant communities providing large amounts of accessible nectar and with intermediate levels of functional diversity were found to be the most likely to enhance the conservation biological control of diverse crop herbivores.

## Introduction

Plant taxonomic and functional diversity promotes interactions at multiple trophic levels and contributes to the multifunctionality of provisioning, supporting, regulating and cultural ecosystem services^1,2^. The relationship between plant functional traits and productivity and soil fertility services has been investigated^2,3^, but its shape remains largely unknown. The level of plant functional diversity maximizing or optimizing the regulating ecosystem services provided by multitrophic interactions also remains unclear.

In crop-dominated landscapes, species-rich habitats, such as linear spontaneous field margins and sown wildflower strips, support populations of arthropods, including predators, parasitoids and pollinators, which are beneficial to the diversity of all taxonomic groups^4,5^. Field margins provide refuges that protect populations of predators and parasitoids against disturbances in the vicinity of cropped habitats, together with the trophic resources (nectar, pollen, alternative preys) required for completion of their life cycles^6^. However, only a few studies have investigated how plant communities could be tailored to promote ecosystem services, such as conservation biological control in particular^7^. Most studies to date have focused on flower strips sown with a single plant composition, but a small proportion have compared mixtures (eight of the 40 studies reviewed by Haaland et al.^8^). As a result, our understanding of the interactions between plant and animal communities remains limited, and attempts to implement or manage habitats to enhance functional biodiversity have not necessarily resulted in a significant decrease in crop herbivore levels^9^.

Attempts to develop strategies for the widespread deployment of conservation biological control face two main challenges. First, a broader range of herbivores and natural enemies than considered to date must be studied, to deal with the large number of crop herbivores; dedicated species-specific screening approaches are no longer appropriate^10,11^. Second, in field conditions, the hypothesis that resource provision increases the size of natural enemy populations and improves biological control is often not borne out. Attractive plant species may divert herbivores from less attractive species, resulting in lower visiting rates than for single-species stands^12^. The effects of plants on arthropod behavior have mostly been analyzed in pure stands, and little is known about the effect of plant assemblages. Interactions may also occur within arthropod communities, through competition between flower visitors^10,13^ and intraguild predation^14^.

The role of morphological matching traits is well known in plant–pollinator interactions, such as those involving bees and hoverflies, in which flower visitation and pollination depend on a good match between insect proboscis length and the depth of the structure holding the nectar in the corolla^15,16^. In parasitoids, the effect of nectar provisioning by individual flower resources has also been related to morphological matching between the interacting organisms^17–19^. Other traits are also involved in nectar accessibility (concentration), resource use (flower attractiveness and insect preferences) and effectiveness, which depends on the physiological requirements of the parasitoid^20^. However, unlike pollinators, parasitoids can be opportunist floral visitors (especially Hymenoptera) making use of an available resource without contributing to pollination. This raises questions about whether trait matching with flowers is as strong in parasitoids as in pollinators.

We developed a trait-matching approach for studies of a community of several parasitoids of five oilseed rape and faba bean herbivores. Upscaling this approach to community level is challenging, because it raises new questions. The amount of flower resources would be expected to maximize the activity of a given parasitoid species, but the diversity of these resources may be more important when considering several parasitoid species. An increase in plant species richness and/or functional diversity may provide more niches for their consumers, promoting niche complementarity and lowering competition, thereby leading to an increase in the diversity of consumers, particularly if the species concerned have different ecological requirements^21,22^.

However, this hypothesis was not supported by recent studies analyzing the effects of the functional diversity of flower resources on predators, parasitoids and pollinators^23,24^. This is not particularly surprising, because species and/or functional diversity is correlated with species and/or functional evenness. Evenness reduces dominance effects, by diluting the relative contribution of the dominant species within the community. For example, mixing plant species on the basis of their flowering periods, to ensure that flower resources are supplied throughout the year, decreases the density of plants flowering at any one time. At peak flowering, more resources are available in communities with a homogeneous phenology than in communities with a heterogeneous phenology, resulting in a non-monotonous relationship between the diversity of resources and their instantaneous availability.

In summary, there is no consensus concerning the relative importance of community species composition and functional structure^25^ to multitrophic interactions and ecosystem functioning^15,26^. Our objective was to distinguish between the respective influences of plant taxonomic richness, functional composition and functional diversity on the functioning of insect communities, studied here by evaluating the effects of parasitoids on a multi-pest community. We specifically addressed the three following questions.

First, is a trait-based approach suitable for predicting the effect of plant assemblages, via the provision of trophic resources, on the parasitism of a community of crop herbivores by several parasitoids? Nectar is an important energy source in the diet of parasitoids. We therefore hypothesized that traits related to temporal and morphological matching between plants and parasitoids can be used to predict the level of crop herbivore parasitism.

Second, over and above the effect of plant functional composition, is there an additional effect of plant taxonomic richness and functional diversity? It was initially hypothesized that an increase in plant species and functional diversity, by providing more niches, would decrease competitive interactions between floral visitors, with a positive effect on parasitoid activity. Alternatively, increases in plant diversity may attract more diverse insect visitors to flowers^27^, with potential negative interactions between them^13^, leading to lower rates of flower visitation by parasitoids^27^ and, ultimately, a lower rate of herbivore parasitism.

Third, how do the respective roles of plant taxonomic and functional structure change when upscaling from a single to several functions (i.e. when considering parasitism at community level)? We expected the functional diversity of plant assemblages, through the provision of diversified resources, to make a more important contribution than functional composition (amount of trophic resources) to the multifunctional effect of parasitoids. We also expected the highest multi-species parasitism levels to be reached at intermediate levels of functional diversity, because, as explained above, a high level of functional diversity may decrease the dominance effects of nectar-providing plants, by diluting the relative contribution of the dominant species within the community.

**Figure 1:**
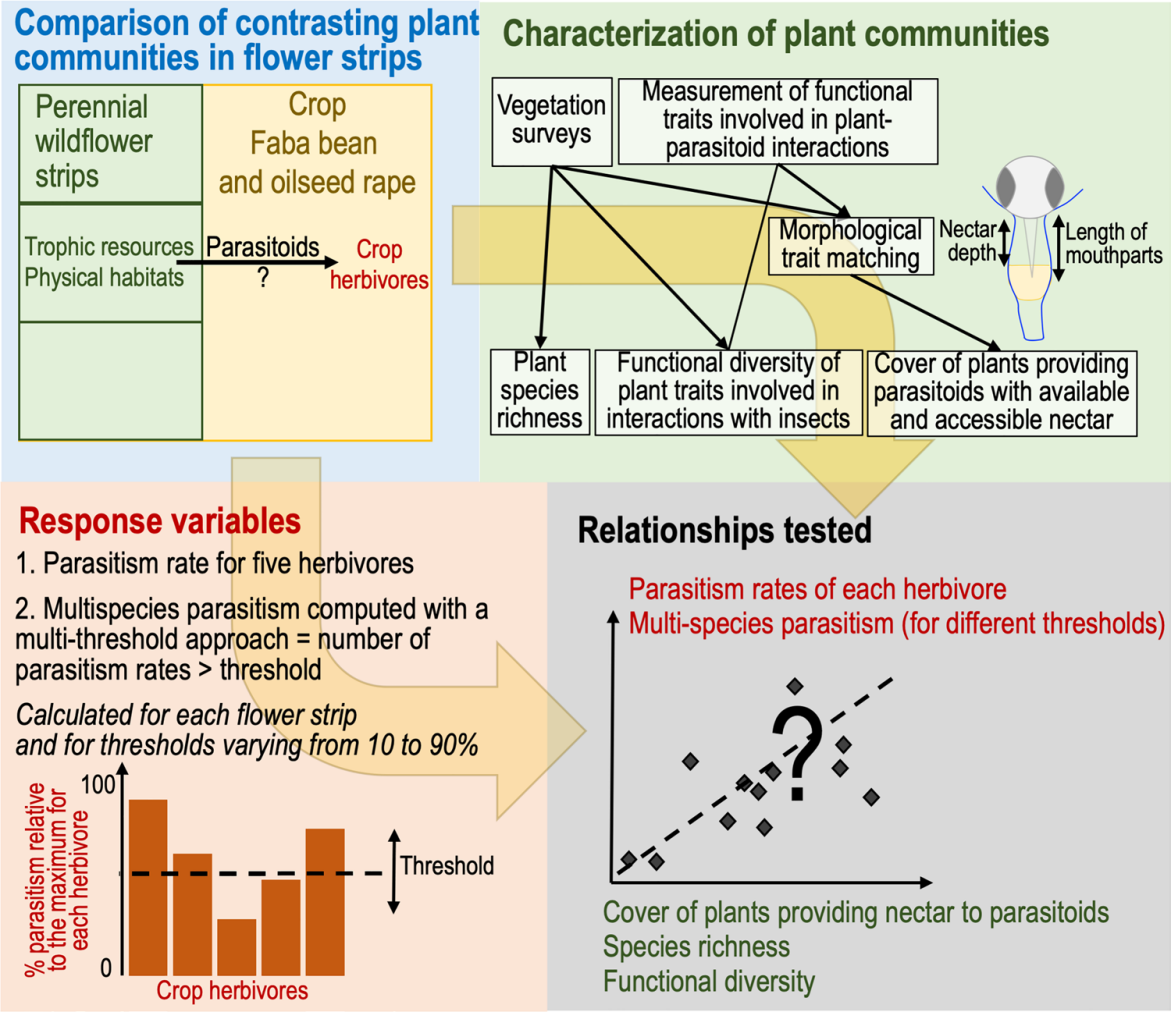
Graphical abstract of the main experimental and methodological approaches used in this study. This figure was created using Microsoft Powerpoint version 16.16.14 (https://www.microsoft.com).

**Figure 2:**
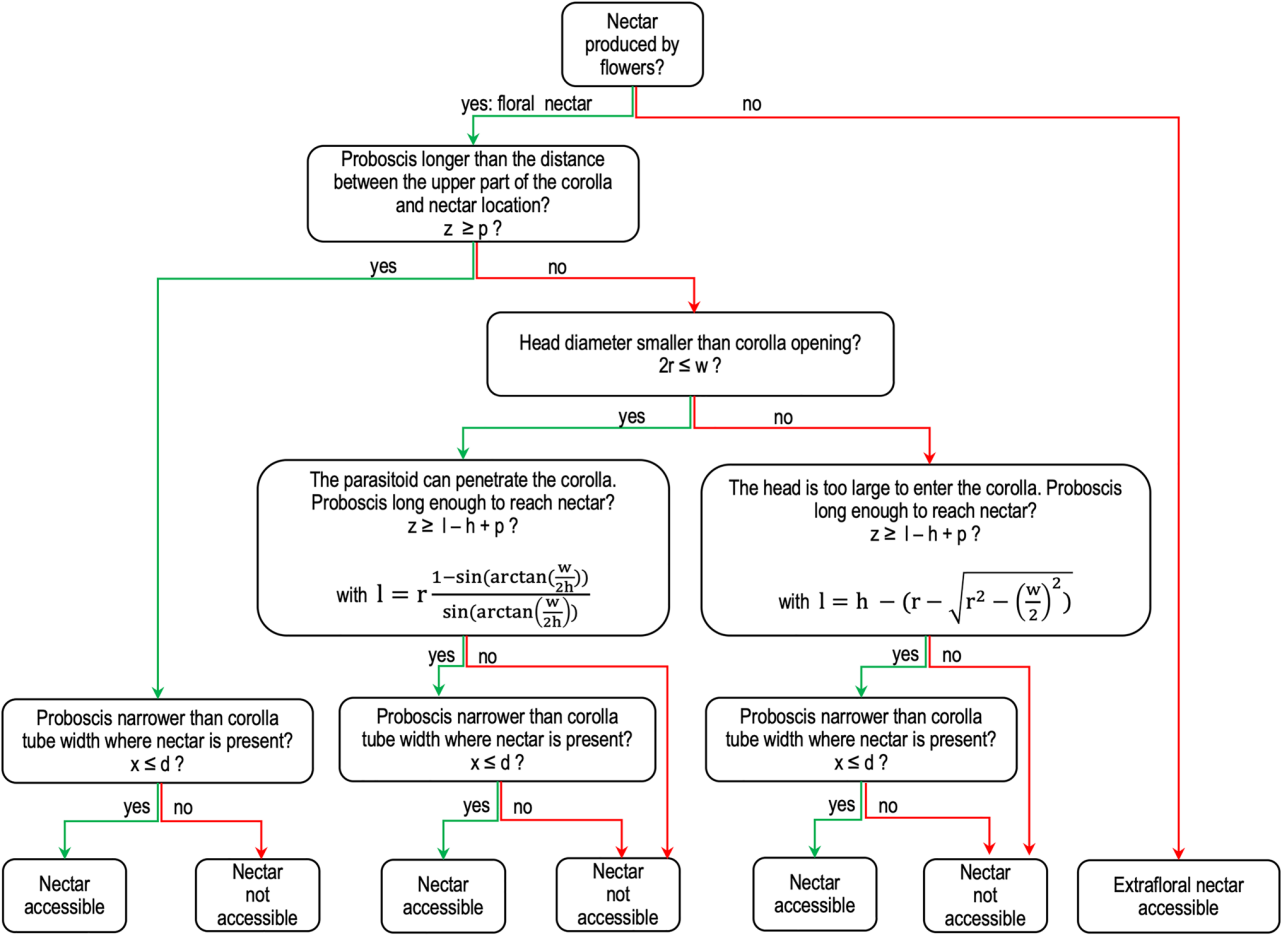
Decision tree used to determine the accessibility of nectar to insects as a function of insect traits (radius of insect head *r,* proboscis length *x* and proboscis width *z*) and flower traits (width of flower opening *w*, corolla height *h*, nectar depth *p*, nectar tube diameter *d*). More information can be found in the Supplementary methods. This figure was re-drawn from Zucchetta^29^, and it was created using Microsoft Powerpoint version 16.16.14 (https://www.microsoft.com).

We tested these hypotheses in a factorial field experiment, in which we compared eight plant assemblages differing in terms of taxonomic composition, species richness and functional diversity (Fig. 1). In the adjacent crop, 5 and 20 m from the wildflower strip, we measured the parasitism of five herbivorous pests of faba bean (in 2016) and of oilseed rape (in 2017). Biological control is important in these crops, as both have a high insecticide treatment frequency index^28^. We investigated the effects of the plant assemblages on parasitism rate, by characterizing the plant cover producing available and accessible nectar. Nectar was considered to be available when it was produced during the period of parasitoid activity. Floral nectar accessibility depended on morphological matching between plant and parasitoid traits measured for each parasitoid and plant within the assemblage. We took into account the ability of the insect to penetrate the flower and its ability to reach the nectar, which depended on corolla shape, nectar depth, insect head size and the size of the mouthparts (Supplementary methods and Fig. 2). As explanatory variables, we also included species richness and the functional diversity of the plant traits involved in plant–insect interactions (traits related to the provision of trophic resources, the temporal availability of these resources, flower attractiveness, nectar accessibility and the provision of physical habitats). Finally, we assessed the ability of the assemblages to contribute to the parasitism of all five herbivores—multi-species parasitism—by evaluating whether high levels of parasitism occurred simultaneously for all herbivores and by determining the degree to which parasitism levels were dependent on the taxonomic and functional composition of assemblages.

## Results

The pressure of herbivore populations in oilseed rape and faba bean was high due to the absence of insecticide treatment (Supplementary results). In general, parasitism rates, averaged over all assemblages, varied from 0.11 ± 0.08 for the herbivore *Brassicogethes aeneus* to 0.45 ± 0.10 for *Bruchus rufimanus*. Parasitism involved a single parasitoid species, except for *Dasineura brassicae,* in which we found three morphospecies (Supplementary Table S4). We first investigated the effects of the different plant assemblages on the rates of parasitism for the five herbivorous species. When we compared parasitism rates across distances, no general distance effect was detected (Supplementary Table S5), other than in *Bruchus rufimanus,* for which parasitism rates were slightly higher at 20 m than at 5 m (Supplementary Fig. S3). In analyses of parasitism rates separately for each distance, the type of assemblage significantly influenced parasitism rates in *B. rufimanus* (*p* value < 10^−4^), *Ceutorhynchus pallidactylus* (*p* value = 0.009) and *B. aeneus* (*p* value = 0.010) 5 m away from the flower strips, and those in *B. rufimanus* (*p* value < 10^−4^) and *Psylliodes chrysocephala* (*p *value = 0.040) 20 m away from the strips (Supplementary Table S6). The effects observed depended on the herbivore species considered (Supplementary Fig. S3).

**Figure 3:**
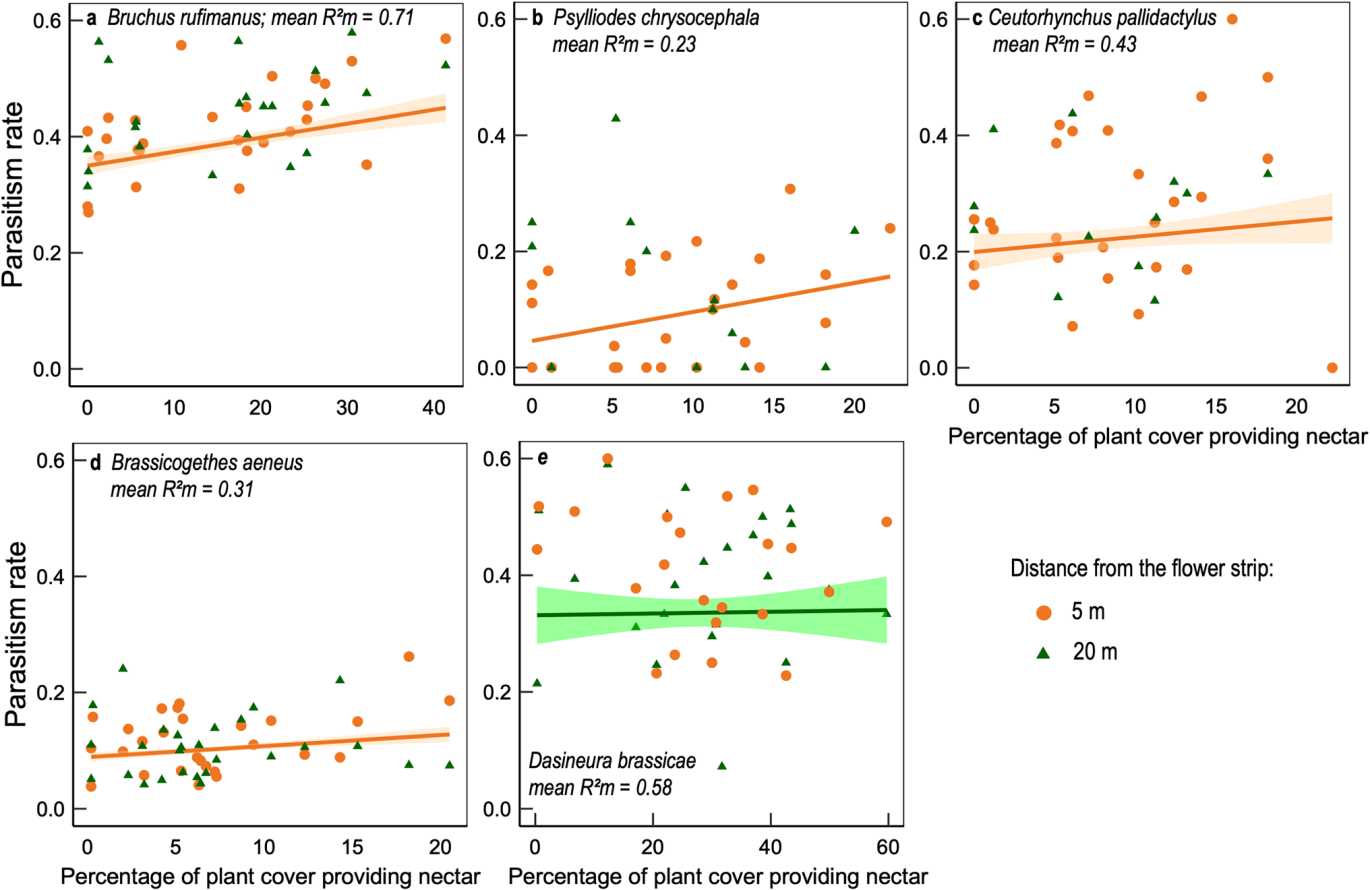
Effect of the percentage of the plant cover providing accessible nectar to parasitoids on parasitism rates in five crop herbivores (mean prediction and 95% confidence intervals). Partial regression plots result from a generalized mixed model, assuming the errors to be binomially distributed. Parasitism rates were measured at 5 m (orange circles) and 20 m (green triangles) from the flower strip. Regressions are shown only when a significant effect nectar resources, is found. The R^2^ are the mean values for the best models detailed in Table S8. This figure was made using R version 3.6.3^30^ (https://www.R-project.org/).

Plant communities comprised 85 species, 41 of which belonged to the 55 sown species, accounting for 92.8% of total plant cover. As the composition of plant communities in 2016 and 2017 did not fully reflect the sown mixtures (Supplementary Fig. S4), we then investigated the effects of the observed plant assemblages by characterizing three explanatory variables: (1) plant species richness, (2) the percentage of plant cover providing available and accessible nectar to parasitoids, estimated by a species trait-matching approach and (3) the functional diversity of the plant traits involved in plant–insect interactions. Sixteen to 49 plant species produced nectar available during the period of parasitoid activity, and the trait-matching approach indicated that the nectar was accessible for only 13–36 of these species, depending on the parasitoids considered (Supplementary Table S7). Statistical models of parasitism rates including trophic resources, number of species and functional dispersion as explanatory variables (individual best models in Supplementary Table S8) were much more parsimonious, with a lower AIC than those including type of assemblage as an explanatory variable (Supplementary Table S6). At a distance of 5 m from the strips, the percentage of plant cover providing nectar, alone or in interaction with species number, had a significant positive effect on parasitism rates in all species except *D. brassicae* (averaged models in Table 1, full version in Supplementary Table S9, Fig. 3). The percentage of plant cover providing nectar was generally the variable with the highest relative importance, and was therefore included in most of the best models. At a distance of 20 m from the strips, parasitism in *B. rufimanus* was negatively affected by a quadratic effect of functional dispersion, and parasitism in *B. aeneus* decreased with the number of species (Table 1).

**Table 1:** Effects of the composition and structure (proportion of plant cover providing accessible nectar, species richness and functional dispersion) of the flower strip plant communities on the rates of parasitism in five herbivorous crop pests and on global multi-species parasitism (quantified via a multi-threshold approach).

Parasitism at 5 m from the strip	Cond. averaged model		*P* (> z)	Weight	Parasitism at 20 m from the strip	Cond. averaged model		*P* (>|z|)	Weight
Explanatory fixed variables	Effect ± SE	z-value			Explanatory fixed variables	Effect ± SE	z-value		
***Bruchus rufimanus* (*n* = 27)**					***Bruchus rufimanus* (*n* = 27)**				
Functional dispersion^2^	− 0.071 ± 0.029	2.286	**0.02**	1	Functional dispersion^2^	− 0.108 ± 0.048	2.185	**0.02**	0.83
Nectar resources	0.087 ± 0.032	2.610	**0.009**	1	Func. disp. × Sp. number	− 0.072 ± 0.032	2.112	**0.03**	0.05
***Psylliodes chrysocephala* (*n* = 27)**					***Psylliodes chrysocephala* (*n* = 12)**				
Nectar resources	0.382 ± 0.166	2.183	**0.02**	0.78	No significant effect				
***Ceutorhynchus pallidactylus* (*n* = 27)**					***Ceutorhynchus pallidactylus* (*n* = 13)**				
Species number	0.214 ± 0.108	1.878	0.06	1	No significant effect				
Nectar res. × Sp. number	0.585 ± 0.271	2.027	**0.04**	0.11					
***Brassicogethes aeneus* (*n* = 27)**					***Brassicogethes aeneus* (*n* = 27)**				
Nectar resources^2^	0.111 ± 0.045	2.367	**0.01**	1	Functional dispersion	0.315 ± 0.159	1.895	0.06	0.86
					Species number	− 0.313 ± 0.143	2.085	**0.03**	0.92
***Dasineura brassicae* (*n* = 25)**					***Dasineura brassicae* (*n* = 26)**				
No significant effect					Func. disp. × nectar res	0.437 ± 0.194	2.107	**0.03**	0.10
**Multi-species parasitism (*n* = 243)**					**Multi-species parasitism (*n* = 117)**				
Threshold	− 1.695 ± 0.188	8.977	**< 10**^**−4**^	1	Threshold	− 1.737 ± 0.269	6.580	**< 10**^**−4**^	1
Functional disp.^2^ × threshold	− 0.289 ± 0.036	2.114	**0.03**	1	Nectar resources	− 0.129 ± 0.075	1.708	0.09	1

Significant P-values are written in bold.

All possible combinations of the plant community variables (nectar resources, species richness and functional diversity, with both linear and quadratic effects) and their interactions were compared.

The best models were ranked according to their AIC (Supplementary Table S5) and we present the results for the averaged best models.

Generalized linear mixed effect models were used, assuming a binomial (parasitism rates) or Poisson (multi-species parasitism) distribution, with strip as a random effect.

All explanatory variables were scaled.

For readability, we report here the results for significant effects only or for variables with a relative importance (weight) greater than 0.70.

The full results are provided in Supplementary Table S9. “n” is the number of observations for each response variable.

We tested the validity of the trait-matching approach, by constructing a neutral model in which the ability of plants to provide accessible nectar was randomly selected rather than determined by trait-matching (for all species or flowering species only). The AIC of neutral models was systematically higher than that of trait-matching models (Supplementary Table S10), except for *P. chrysocephala* at 20 m from the strips, for which the nectar resource effect was not significant. This indicates that estimating nectar accessibility via the trait-matching model provides a better predictor than selecting interactions randomly.

We used a multiple-threshold^31^ approach to assess the ability of the assemblages to support the parasitism of several herbivore species simultaneously, as a contribution to ecosystem multifunctionality. Rather than averaging non-substitutable parasitism rates, we calculated, for each treatment, the number of herbivores for which the parasitism rate exceeded a given proportion, or threshold, of the maximum parasitism rate observed. We plotted this multi-species parasitism against the type of assemblage, for various threshold values (Supplementary Fig. S5). We found that the number of pest parasitism rates simultaneously reaching a given threshold depended on the threshold chosen. For low thresholds (e.g. 10%), parasitism rates reached 10% of their maximum values for four or five herbivores in all assemblages. Conversely, parasitism rates never reached 90% of the maximum value for more than two species in any assemblage, suggesting that they could not be maximized for all herbivores simultaneously. Defining a specific threshold for determining whether a given parasitism rate is sufficiently high to contribute to herbivore parasitism would be arbitrary. We therefore calculated multi-species parasitism for a range of thresholds varying from 10 to 90% of the maximum parasitism rate for each herbivore, in steps of 10%. We then took the threshold variable (continuous) into account in the regressions carried out with the dataset compiling multi-species parasitism determined at each threshold. Diversity effects might also be expected to be stronger when high thresholds are imposed to achieve multifunctionality, with a larger number of plant species or plant functions required to maintain the parasitism rates at high levels for several pests^31^. We therefore tested the interaction between the threshold variable (nine values) and the other variables describing plant communities in the regressions.

**Figure 4:**
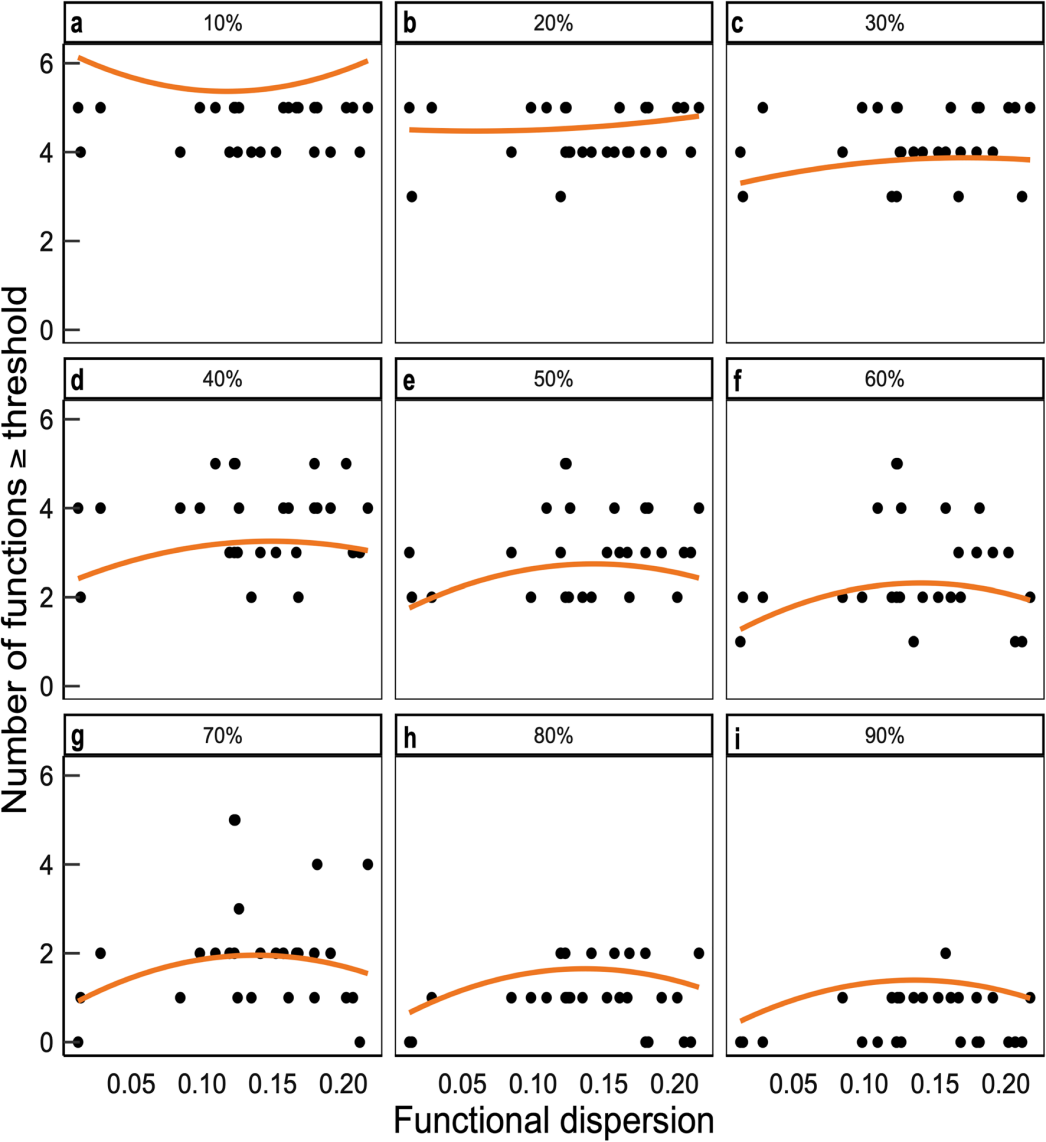
Partial regression plots showing the relationship between the functional dispersion of plant communities (based on traits involved in plant–parasitoid interactions) and the multi-species parasitism of five herbivorous crop pests, measured at a distance of 5 m from the flower strips. Multi-species parasitism, quantified with a multi-threshold approach, is the number of herbivore species for which the rate of parasitism exceeds a given percentage (thresholds from 10 to 90%) of the maximum parasitism achieved for each species. This figure was made using R version 3.6.3^30^ (https://www.R-project.org/).

When the plant communities were described in terms of their observed functional composition and structure, rather than assemblage types, multi-species parasitism at a distance of 5 m from the strips was significantly influenced by functional dispersion (quadratic term) in interaction with the threshold value (*p* value = 0.035). This predictor had, by far, the highest relative importance, with a weight of 1 (Table 1, Supplementary Table S9). This positive interaction implies that functional dispersion has a larger effect at high threshold values, i.e. in analyses of the ability of plant communities to support high rates of parasitism for all herbivore species simultaneously. At threshold values above 30%, plant functional dispersion had a hump-shaped effect, with multi-species parasitism levels highest at intermediate functional dispersion values (Fig. 4). At a distance of 20 m from the strips, plant community structure had no detectable effect on multi-species parasitism (Table 1, Supplementary Table S9, Supplementary Fig. S6).

We repeated the statistical analyses shown in Table 1 on two subsets of assemblages, to disentangle the effects of sown species richness from those of sown functional diversity more effectively: (1) we compared low and high functional diversity while controlling for species richness (using only assemblages with a medium species richness) and (2) we assessed the role of species richness across plant assemblages with high functional diversity (detailed results in Supplementary Table S11). In general, when analyzing the individual parasitism rates, the nectar resources variable was still among those with the highest Akaike weight, even though most effects were no longer significant for the reduced dataset. At 5 m from the strips, the functional dispersion effect on multi-species parasitism was no longer significant, but this variable still had a high Akaike weight (0.75). Controlling for sown functional diversity revealed no underlying effect of species richness.

## Discussion

We investigated the effects of the species richness, functional composition and functional diversity of plant communities, which we manipulated experimentally, on the ability of these communities to support biological control of the five herbivorous crop pests considered. We disentangled these effects by considering them within the same analyses, as proposed by Dias et al.^32^. We found that the percentage of plant cover providing available and accessible as a resource to parasitoids, estimated with a species trait-matching approach, was the factor best explaining parasitism rate in four out of the five herbivorous species considered, and that plant functional diversity was the most important factor when all the species were considered together. A limitation of the experimental scheme is that the initial gradients of sown species richness and sown functional diversity are not fully orthogonal. We took this into account by analyzing the observed richness and functional diversity as continuous variables that were only partly correlated.

The type of plant assemblage initially sown did not account for parasitism rates, despite the differences in species richness and functional diversity between assemblages. We therefore developed a mechanistic trait-matching approach, as previously described for pollinators e.g.^33^ or Syrphidae in particular^16^. We adapted this approach to the smaller hymenopteran parasitoids, which have very short or unspecialized mouthparts, to assess the percentage of plant cover providing available and accessible nectar as a resource in plant communities.

At a distance of 5 m from the flower strips, increasing amounts of this trophic resource increased parasitism rates in four of the five herbivorous pests studied (exception: *Dasineura brassicae*). Moreover, mechanistic models performed better than neutral models in which interactions were randomly selected. This validates our first hypothesis that traits related to temporal and morphological matching between plants and parasitoids can be used to predict the level of parasitism. In general, nectar resources promote parasitoid longevity, host-searching ability and fecundity^34^, potentially accounting for the higher rates of parasitism observed. These findings demonstrate the relevance of a mechanistic approach, based on trait-matching between plants and parasitoids, for investigations of the ways in which plant communities modify the functioning of insect communities. However, pairs of matching traits cannot predict all species interactions correctly^33^. For example, we found that estimated floral or extrafloral nectar resources had no effect on parasitism in *Dasineura brassicae*. There are several possible reasons for this: (1) the parasitoids of this species may be pro-ovigenic, with adults emerging with sufficient nutrient reserves to ensure that they have little or no need to find food^35^, (2) the parasitoids of this species may rely on other hosts in the flower strips, on other trophic resources, such as pollen or honeydew, or may rely on host feeding^36^, (3) the parasitoids may have fed on oilseed rape flowers, which could have provided a massive amount of nectar resources in April and May, (4) other traits may have prevented the use of nectar or (5) a patchy distribution of the host population may have masked the effect of the different flower strips.

In our estimation of nectar resources, we took into account (1) the temporal match between periods of nectar production and insect flight activity and (2) morphological constraints on the size of the insect head and mouthparts imposed by flower shape. These topological traits sensu Gravel et al.^37^ determine the occurrence of trophic interactions. The intensity of these interactions also depends on traits relating to flower attractiveness or the nutritional characteristics of the nectar^6^, which were not taken into account here. Parasitoid preferences for particular flower resources and the effects of these resources on insect fitness can be determined^38^, but the traits underlying these interactions are not straightforward to identify or easy to measure. These interactions may be highly specific, and a trait-based approach is not necessarily more straightforward than a species-based approach.

Previous studies have reported positive correlations between the diversity of nectar resources and the diversity of pollinators^21,39^, and between the diversity of nectar resources and the frequency of plant–pollinator interactions^40^. We, thus, expected plant taxonomic richness and functional diversity to affect parasitism rates. However, after accounting for the effect of nectar resources, we found no clear additional effect of species richness or functional diversity on the rate of parasitism in each species. In general, these variables were either non-significant or had a very low Akaike weight in the best statistical models. This suggests that increases in plant taxonomic richness and functional diversity in our experiment did not improve the exploitation of the available flower resources by the seven studied parasitoids through niche partitioning with other flower-visiting insects. These resources may not have been a limiting factor in our experiment, or there may have been no negative interactions, as in competition between flower-visiting insects.

One notable exception to this general finding was the negative effect of plant functional diversity on the rate of *B. rufimanus* parasitism at distances of 5 and 20 m from the flower strips. This relationship may result from the emergence of negative interactions between floral visitors in functionally diversified plant communities. Indeed, Campbell et al.^13^ previously reported that rates of flower visitation by parasitoids were highest in stands of plants bearing only flowers with short corollas, and that these rates were much lower in plant stands bearing flowers with more diverse traits, due to higher levels of competition with bumblebees and hoverflies. Another hypothesis would be that greater functional diversity would have resulted in a higher abundance of predators targeting adult parasitoids^41^. Unfortunately, in the absence of data on flying parasitoids, we cannot address this hypothesis here.

We then considered multi-species parasitism, by assessing the ability of the strips to support the parasitism of all five herbivorous pests simultaneously. None of the strips maximized parasitism rates for all five species simultaneously, demonstrating the existence of a trade-off between these individual biological control services. This trade-off may be related to the high degree of trophic specialization of the parasitoids^42^, the short period of time for which they are active, and their ability to feed on only a very small number of plant species. Plant assemblages providing abundant flower resources for a given parasitoid (e.g. because they flower early in the season, in March and April) did not provide such abundant resources for other parasitoids (e.g. resources provided by plants flowering a few months later). Strip size and connectivity with semi-natural habitats may also have been insufficient to facilitate colonization of the experimental sites by a diverse community of parasitoids.

As a result, multi-species parasitism close to the strips was not dependent on total nectar resources. Instead, multi-species parasitism was explained by the functional diversity of the plant communities, with multifunctionality levels highest at intermediate levels of plant functional diversity. However, the effect of functional diversity did not remain significant after controlling for initial species richness in the sown assemblages, even though functional diversity still had the highest relative importance in the models. At low levels of plant functional diversity, an increase in plant functional diversity may provide the parasitoid community with more potential niches, thereby increasing parasitoid species richness, as predicted from knowledge of plant–pollinator interactions^21^, thus increasing the level of multi-species parasitism. However, too high a level of plant functional diversity may dilute the expression of the trait combinations supporting each type of parasitoid, including the nectar resources associated with each feeding niche, thereby decreasing parasitism rates at the level of the community of herbivorous pests.

In general, the nectar resources or functional diversity of the various plant communities had little or no effect 20 m away from the flower strips. This does not imply an absence of spillover at this distance, because parasitism rates were generally similar at 5 and 20 m. Instead, it reflects a limitation of our experimental design. The treatments were close together and the distance to the different types of strip was more similar in the central parts of the field. We cannot exclude the possibility of confounding effects between the different measurements of plant community composition and diversity.

These results, obtained with a community of specialized insects, are complementary to those previously obtained for a generalist-dominated pollinator community^27^, in which pollinator species richness was not affected by plant functional diversity. Our results are consistent with previous findings of a lack of association between plant functional diversity and the abundance and diversity of natural enemies^7,23,43^. Using a broad gradient of plant functional diversity and accounting for mass-ratio effects simultaneously (quantification of nectar resources), we were able to demonstrate the existence of a hump-shaped relationship between plant functional diversity and biological control by parasitoids in a two-crop rotation.

## Conclusion

The idea that plant taxonomic and functional diversity promotes interactions at higher trophic levels, with a positive effect on ecosystem functions, is increasingly being challenged. We used a trait-based approach to clarify the relationships between plant and insect communities. We found a positive effect of nectar resources on the functions performed by parasitoids, confirming the predominant role of mass-ratio effects. We also obtained evidence for an effect of functional diversity only when the ability of plant communities to support the simultaneous biological control of the five herbivores studied was considered. Multi-species parasitism was maximal at intermediate levels of functional diversity, and plant species richness had a negligible influence relative to functional metrics.

Based on trait-matching between plants and insects, we were able to establish a powerful mechanistic link between the functional structure of plant communities and the functions delivered by insect communities, in this case, parasitism rates in several herbivorous crop pests. Matching traits have already been shown to be relevant for explaining food web interactions^33,44^ and community structure^45^, but their cascade effects on ecosystem services have less frequently been reported. The generalization of this approach will be powerful for the description, from a functional perspective, of the contribution of natural and semi-natural habitats to ecosystem services^46^.

Most previous studies on biological control did not focus on crops with high estimated intensities of insecticide use^47^. Here, we focused on herbivorous pests of oilseed rape and faba bean, which have been little studied to date, despite causing major losses of crop yield and quality^48,49^. Another original feature of this study was its evaluation of the ability of flower strips to affect the biological control of several herbivores simultaneously, in a “multi-pest community” approach. Our results provide useful insights into the design of perennial plant mixtures for creating or restoring habitats supporting natural enemies. Plant communities providing large amounts of accessible nectar resources and with intermediate levels of functional diversity are the best able to enhance the conservation biological control of diverse crop herbivores.

## Materials and methods

### Design of species assemblages with contrasting species and functional diversities

We designed eight assemblages of native and perennial plants differing in terms of species richness (three levels), functional diversity of the traits involved in plant–arthropod interactions (two levels) and species identity (two sets of species). We combined these first two factors to define four categories of plant assemblages for further study:

- Low functional diversity and medium species richness (14 species), LFMS;
- High functional diversity and low species richness (9 species), HFLS;
- High functional diversity and medium species richness (14 species), HFMS;
- High functional diversity and high species richness (29 species), HFHS.

For each of these four categories, we designed two assemblages with different species identities, as described in the Supplementary information, resulting in eight plant assemblages in total. Functional characterization was based on a rough classification of plant species into functional groups (Supplementary Table S1), according to the mains traits involved in plant–species interactions easily accessible from databases: (1) flower resources, i.e. floral and extrafloral nectar or pollen, (2) accessibility of the resource, depending on flower shape, (3) availability of the resource, i.e. the flowering period and (4) flowering height.

We generated the seed mixtures from commercial seeds, using ecotypes of local origin wherever possible (northern part of the Parisian basin, France). All applicable international, national, and institutional guidelines relevant for the use of plants were followed.

### Experimental design

The experiment was conducted between 2013 and 2017 in a 6.5-ha field at Grignon, France (N 48.837, E 1.956), on a deep loamy clay soil, in which soil depth decreased along a gradient from north to south. The field was divided in three blocks running from north to south to take this soil heterogeneity into account.

Each assemblage was sown on a 6 × 44 m^2^ strip, with three replicates (Supplementary Fig. S2), with each assemblage represented once per block. A control treatment, sown with the same crop species as the rest of the field, was also included in the experimental design, resulting in nine experimental treatments in total. From the autumn of 2013 to the 2017 harvest, a winter barley–maize–faba bean–oilseed rape rotation was grown in the field. Crops were managed without insecticide treatment, but with a mean of 0.75 fungicide and 1.25 herbicide treatments per year. The observations were made in faba bean in 2016 and in oilseed rape in 2017.

### Botanical assessments and functional characterization of the plant communities

Botanical assessments were conducted in April and June, in 2016 and 2017. In each treatment, the vegetation was assessed in 3 × 15 m^2^ plots at a position representative of the whole strip, generally in the center of the strip, to prevent edge effects. The percentage of the ground covered by each sown or spontaneously growing plant species was estimated by eye, by the same observer in each case. We noted the phenological development stage of each species in each treatment on an 11-point scale, to ensure an accurate assessment of flowering phenology. In the control plots (sown with the crop species only), we took into account the resources provided by weed species.

The functional characterization of plant communities was based on the plant traits assumed to be involved in plant–parasitoid interactions^6^ (Supplementary Table S3). These traits were related to (1) the provision of trophic resources (presence of floral and extrafloral nectar, qualitative estimation of floral nectar), (2) the temporal availability of the resource (date of flowering onset and duration of flowering), (3) flower attractiveness (flower or inflorescence diameter, color, UV reflectance pattern), (4) nectar accessibility (flower opening diameter, corolla height, nectar depth and nectar tube diameter) and (5) the provision of physical habitats (leaf distribution, vegetative and flowering height). We measured most of these traits, particularly all those relating to flower morphology, phenology and nectar provision (see more detailed methods in the Supplementary information). Only a few were retrieved from previous publications and online databases: flower color and UV reflectance pattern, leaf distribution, vegetative and flower height.

These traits were used (1) to determine the accessibility of nectar to each parasitoid (see below) and (2) to calculate the functional diversity of the plant assemblages. We calculated functional dispersion as the abundance-weighted mean distance of individual species from the centroid of all species in the trait space^50^ and Rao quadratic entropy^51^. Since these two parameters were highly correlated (Supplementary information), we considered only functional dispersion a measurement of functional diversity. The traits associated with the provision, availability and accessibility of nectar resources were measured for all the dicotyledonous species sown and for all spontaneous species occurring in the plant communities and flowering during parasitoid activity. Overall, considering the traits we measured and those retrieved from databases, the trait matrix was complete for more than 95% of the species, accounting for 99.6% of total plant cover.

### Assessment of the levels of parasitism on five herbivorous pests of faba bean and oilseed rape

In the adjacent crop, 5 and 20 m from the wildflower strip, we measured the level of parasitism in one herbivorous pest of faba bean (2016) and four herbivorous pests of oilseed rape (2017). We chose a distance close to the strip (5 m) to prevent confounding effects with the other adjacent strips, knowing that their effect is the strongest in the first few meters from the strip^52^. A further distance was also chosen (20 m) to determine whether the strips promoted biological control at field level, while taking into account the spatial constraint of the distance between strips (50 m between opposing strips).

All the protocols are detailed in the Supplementary information. Parasitism was assessed in *Bruchus rufimanus* larvae after the visual examination of faba bean seeds after harvest. For oilseed rape, we collected and reared *Ceutorhynchus pallidactylus* and *Psylliodes chrysocephala* larvae until the adult stage or parasitoid emergence. In *Brassicogethes aeneus* larvae, parasitism was assessed by observing the eggs of *Tersilochus heterocerus* in the host larvae in oilseed rape flowers. Finally, after oilseed rape harvest, we retrieved cocoons of *Dasineura brassicae* from the soil, which we dissected, recording the number of cocoons occupied by parasitoids.

### Measurement of parasitoid traits

We carried out morphological measurements on parasitoids (Supplementary Table S4), to determine their degree of access to the nectar provided by plants, as a function of the size of their mouthparts and head, which limit corolla penetration, using an approach analogous to that of van Rijn and Wäckers^16^. Parasitoid individuals, preserved in 70% ethanol, were obtained (1) from our rearing experiments (for *Bruchus rufimanus*, *Psylliodes chrysocephala* and *Ceutorhynchus pallidactylus*), (2) from the dissection of cocoons for *Dasineura brassicae* or (3) by field sampling in the flower strips with a sweep net in April 2017 to collect *Tersilochus heterocerus*, parasitoids of *Brassicogethes aeneus* identified with^53^. For each parasitoid species or morphospecies, we measured, on at least 10 individuals, proboscis length, proboscis width (at mid-length)^54^ and the maximum dorsal head width, including the eyes. Observations were carried out under a binocular microscope (Leica M80, 60 ×) linked to a video camera (Moticam 10, Motic), and measurements were made with ImageJ v1.50i digital image analysis software (National Institute of Health, Bethesda, http://imagej.nih.gov/ij).

### Nectar resources for parasitoids

We estimated the amount of nectar provided by the plants by summing, for each flower strip corresponding to a treatment, the percent cover of plants providing available and accessible nectar, as assessed in vegetation surveys. Separate estimates were obtained for each parasitoid species or morphospecies.

Plant species producing floral or extrafloral nectar were first selected on the basis of the observations detailed in the botanical assessment section. Nectar was considered to be available when it was produced during the period of parasitoid activity (Supplementary Table S4), by selecting species at the flowering stage or producing extrafloral nectar based on the phenological observations carried out during the botanical assessments. Nectar accessibility depended on morphological matching between plants and insects. Extrafloral nectar, which is not enclosed in a perianth, but produced on bracts or stipules, was considered to be accessible. We determined the accessibility of floral nectar with a mechanistic trait-based approach (Supplementary Information), by adapting the geometric model proposed by van Rijn and Wäckers^16^. A decision tree was built (Fig. 2) to take into account the three constraints limiting nectar accessibility: (1) ability of the insect to penetrate the flower, which is dependent on *head size* and *flower opening*, (2) ability to reach the nectar, which depends on *proboscis length*, *nectar depth* and *corolla height*, and (3) *proboscis width* and *nectar tube diameter in the presence of nectar*.

### Statistical analyses

We investigated the effects of the different plant assemblages on the rates of parasitism for the five herbivorous species, at 5 and 20 m from the flower strip, considered separately as individual response variables. We first tested the effect of each assemblage (nine treatments as factors) on parasitism rates. We used generalized linear mixed models in the lme package^55^, with a binomial error distribution. The models included plot (*n* = 9 flower strips × 3 replicates = 27), strip (1–3) or block (1–3) as a random effect. All models were run three times with each random effect variable, and the model giving the lowest AIC was retained. Strips consistently yielded the lowest AIC. This factor was therefore introduced as a random effect variable for all statistical analyses. The significance of the fixed effects was evaluated by type II analyses of deviance with Wald chi-squared tests from the Anova function from the car package^56^. If a significant effect (*p* value < 0.05) was detected, we performed Tukey-HSD post hoc tests for pairwise multiple comparisons of the estimated marginal means of each treatment with the multcomp package^57^.

We investigated the effects of the plant assemblages on parasitism rates, by characterizing three explanatory variables: (1) plant species richness, (2) percentage of plant cover providing nectar accessible to parasitoids, estimated from the traits relating to nectar availability and accessibility and (3) functional diversity of all the previously described plant traits involved in plant–insect interactions, including attractiveness. These explanatory variables were standardized to account for the large differences in scale between them. We tested our hypotheses with generalized linear mixed models, assuming that the errors followed a binomial distribution. For each response variable (parasitism on each species at each distance), we tested models including, as fixed effects, these three explanatory variables with their linear and quadratic terms (to test for non-linear patterns), and with two-level interactions between the three linear terms. As described above, the strip was introduced as a random effect variable. Using a multimodel inference procedure^58^, we tested models including all possible additive combinations of the nine predictors, ranked according to the Akaike information criterion (AIC), fitted by maximum likelihood methods. Models with a ΔAIC < 2 were selected and we present the statistical results for the conditional averaged model. Akaike weights were calculated to assess the relative importance of each predictor. Only predictors with a weight above 0.70 are interpreted.

We tested the validity of the trait-matching approach, by constructing a neutral model in which interactions were randomly selected rather than determined by trait-matching, whilst keeping constant the total number of plant–parasitoid interactions. We tested (1) the morphological trait-match, by randomly selecting the interacting plant species from those that were in flower during the period of adult parasitoid activity and (2) the temporal and morphological trait-match by randomly selecting interacting plant species from all those present in the plant community, whatever their phenological stage. We then summed, for each treatment, the percent cover of these randomly selected plant species. This new explanatory variable was introduced as an alternative to the cover of plants producing accessible nectar in the statistical models investigating the effects of plant assemblages on the parasitism rates for each herbivore. One thousand iterations were performed for each parasitoid. We averaged the AIC of neutral models and compared them with the trait-matching model.

Finally, we assessed the ability of the assemblages to contribute to multi-species parasitism, by using the multiple-threshold approach to determine whether high levels of parasitism could be simultaneously achieved for several different herbivores^31^. Rather than averaging non-substitutable parasitism rates, the threshold-based approach^31^ involves the calculation, for each treatment, of the number of herbivores for which the parasitism rate exceeds a given threshold (% of the highest observed parasitism rate for each herbivore). Defining a specific threshold for determining whether a given parasitism rate is sufficiently high to contribute to herbivore parasitism would be arbitrary. We therefore calculated multi-species parasitism rates for a full range of thresholds, from 10 to 90% of the maximum parasitism rate (mean of the three highest recorded values), in 10% steps, and we took this threshold effect into account in the regression analyses. In each experimental treatment and for each threshold, the multi-species parasitism rate was calculated as the number of herbivore parasitism rates locally exceeding the threshold value. Diversity effects might also be expected to be stronger for higher proportions of parasitism rates exceeding the threshold value, to reach multifunctionality, i.e. a larger number of plant species or plant functions would be required to maintain several parasitism rates at high levels^31^. We therefore included in the regressions an interaction between the threshold variable and the other variables describing plant communities.

We investigated the effect of the plant assemblages on multi-species parasitism, by first testing the effect of each assemblage separately. Visual examination of the results (Fig. 4) showed that multi-species parasitism was dependent on the chosen threshold: when it was very high, only a few assemblages provided a high parasitism rate (e.g. 90% of the maximum observed value) for all herbivores, whereas a threshold of 50% better discriminated between assemblages. We therefore included in the models the threshold variable (continuous) and its interactions with the other fixed-effect variables. We used generalized linear mixed models with a Poisson error distribution and the strip as a random effect. We assessed the significance of effects as previously described for individual parasitism rates. We then tested the relationships between multi-species parasitism and (1) plant species richness (averaged over the two years), (2) percentage of plant cover providing accessible nectar (averaged for all parasitoids) and (3) functional diversity (averaged over the two years). We used the same approach as previously described for individual parasitism rates. The models included an additional effect of threshold values, introduced as a continuous fixed-effect variable, alone and in interaction with all explanatory variables. We prevented over-parameterization, by applying the model selection procedure only to models containing a maximum of five fixed-effect variables.

We estimated the variance explained by the models with the marginal and conditional pseudo-R^2^ statistic^59^. Diagnostic residual plots for all models were confirmed with the DHARMa package^30^. All statistical analyses were performed with R software, version 3.6.3^60^.

## Supplementary Information

Supplementary Information.

## Data Availability

The datasets generated during and analyzed in this study are available from the Zenodo repository: https://doi.org/10.5281/zenodo.4327257.
