# Supplementary material for: The hump-shaped effect of plant functional diversity on the biological control of a multi-species pest community
Source: Sci Rep. 2021 Nov 4;11:21635. doi: 10.1038/s41598-021-01160-2 (PMC8568967; doi:10.1038/s41598-021-01160-2)
Supplement: Supplementary file 1 — Supplementary Information. [file 41598_2021_1160_MOESM1_ESM.pdf]

Supplementary information

The hump-shaped effect of plant functional diversity on the biological control of a multi-species pest community

Antoine Gardarin, Justine Pigot and Muriel Valantin-Morison

Supplementary methods: Design of species assemblages with contrasting species and functional diversities

We constructed the high functional diversity - medium species richness diversity (HFMS) assemblages by choosing species from each of the 12 functional groups identified in Table S1. The low functional diversity - medium species richness (LFMS) assemblages were obtained by reducing the number of functional groups to seven and increasing the number of species per group, so as to keep species richness constant. In the HFLS assemblages, we retained only the species of the HFMS assemblages belonging the most highly contrasting functional groups, to obtain the highest diversity possible. In the HFHS assemblages, we increased species richness and kept functional diversity as constant as possible by adding extra species with trait combinations closely resembling those already present in the HFMS assemblages.

We constructed the medium species richness - high functional diversity assemblages (HFMS) by choosing one species from each of the nine functional groups identified in Table S1. The low functional diversity assemblages (LFMS) were then obtained by reducing the number of functional groups to seven and by increasing the number of species per group, so as to keep species richness constant. In the HFLS assemblages, we retained only the species of the HFMS assemblages from the most strongly contrasting functional groups, so as to obtain the highest diversity possible. In the HFHS assemblages, we increased species richness and kept functional diversity as constant as possible by adding supplementary species, with trait combinations as similar as possible to those already present in the HFMS assemblages. Thus, at high functional diversity, the increase in species richness was associated with an increase in functional redundancy.

This process was performed for two different lists of dicotyledonous species, resulting in eight different assemblages (Table S2). We added the same three tussock grass species, in similar amounts, to each of the assemblages (Table S2). We selected common and native species and preferred perennial species over annuals, to ensure the durability of the sown communities. Within each assemblage, the species were present in similar proportions, on the basis of seed numbers and the thousand-seed weight of each batch of seed. .

Supplementary Table S1. List of the plant functional groups included in the four types of assemblages (LF and HF indicate low and high functional diversity, respectively, and LS, MS and HS correspond to low, medium and high species richness, respectively). The sources of the trait values are indicated in the Materials and Methods.

Functional group	Flower class according to Müller	Flower type according to Kugler	Month in which flowering starts	Month in which flowering ends	Presence of nectar	Amount of pollen	Extra-floral nectar	Plant height	LFMS	HFLS	HFMS	HFHS
1	Flowers with open nectar	Disk flowers with nectar open	6-7	9	Present	Present	None	Medium height to tall	x	x	x	x
2	Flowers with partly hidden nectar	Disk flowers with nectar ± hidden nectaries at base of stamens	4	6	Present	Present	None	Medium height to tall	x	x	x	x
3	Flower associations with totally hidden nectar	Flower heads, Asteraceae, ray and disk flowers	6	9-10	Present	Present	None	Medium height to tall	x	x	x	x
4	Flowers with open or partly hidden nectar	Disk flowers with nectar open or nectar ± hidden in center of flower	4-5	6-7	Present	Present	None	Medium height to tall	x		x	x
5	Flowers with totally hidden nectar	Stalk disc flowers, stamina and pistil within tube	5	7	Present	Present	None	Medium height to tall	x			x
6	Flower associations with totally hidden nectar	Flower heads, Asteraceae or non-Asteraceae	7	9-10	Present	Present	None	Tall	x		x	x
7	Hymenoptera flowers	Flag blossom, Fabaceae type	6	7 to 9	Present	Present	None	Medium height to tall	x			x
8	Hymenoptera flowers	Flag blossom, Fabaceae type or true lip flowers	4-5	6 to 9	Present	Present	None	Short		x	x	x
9	Flowers with totally hidden nectar	Several types	1 to 3	5	Present	Present	None	Short		x	x	x
10	Flower associations with totally hidden nectar or Hymenoptera flowers	Flower head or flag blossom	5 to 6	7	Present	Present	Present	Medium height to tall		x	x	x
11	Pollen flowers	Pollen flowers	5 to 7	8 to 9	None	Plenty	None	Medium height to tall			x	x
12	Flowers with partly or totally hidden nectar	Disk flowers with nectar ± hidden	5-6	8 to 9	Present	Present	None	Medium height to tall				x

Supplementary Table S2. Composition of the plant assemblages

Assemblages	LFMS1	LFMS2	HFLS1	HFLS2	HFMS1	HFMS2	HFHS1	HFHS2
Initial functional diversity	Low	Low	High	High	High	High	High	High
Initial species richness	Medium	Medium	Low	Low	Medium	Medium	High	High
Species identity list	1	2	1	2	1	2	1	2
Functional redundancy	0.890	0.889	0.836	0.836	0.871	0.875	0.908	0.913
Dicotyledonous species								
Anthriscus sylvestris (L.) Hoffm.	x						x	
Arctium minus (Hill) Bernh.	x						x	
Foeniculum vulgare Mill.	x						x	
Hesperis matronalis L.	x						x	
Leucanthemum vulgare Lam.	x						x	
Medicago sativa L.	x						x	
Securigera varia (L.) Lassen	x						x	
Trifolium pratense L.	x						x	
Achillea millefolium L.	x		x		x		x	
Alliaria petiolata Cavara & Grande	x		x		x		x	
Heracleum sphondylium L.	x		x		x		x	
Cyanus segetum L.			x		x		x	
Trifolium repens L.			x		x		x	
Veronica hederifolia L.			x		x		x	
Centaurea scabiosa L.					x		x	
Euphorbia cyparissias L.					x		x	
Hypericum perforatum L.					x		x	
Tanacetum vulgare L.					x		x	
Verbascum densiflorum Bertol.					x		x	
Ajuga reptans L.							x	
Bellis perennis L.							x	
Capsella bursa-pastoris (L.) Med.							x	
Echium vulgare L.							x	
Galium odoratum (L.) Scop.							x	
Malva sylvestris L.							x	
Potentilla reptans L.							x	
Carum carvi L.		x						x
Cynoglossum officinale L.		x						x
Daucus carota L.		x						x
Hypochaeris radicata L.		x						x
Jacobaea vulgaris L.		x						x
Lotus corniculatus L.		x						x
Onobrychis viciifolia Scop.		x						x
Trigonella officinalis (L.) Lam.		x						x
Barbarea vulgaris R. Br.		x		x		x		x
Cota tinctoria L.		x		x		x		x
Pastinaca sativa L.		x		x		x		x
Medicago lupulina L.				x		x		x
Stellaria media (L.) Vill.				x		x		x
Vicia sativa L.				x		x		x
Cichorium intybus L.						x		x
Galium mollugo L.						x		x
Knautia arvensis (L.) Coult.						x		x
Plantago lanceolata L.						x		x
Verbascum lychnitis L.						x		x
Geum urbanum L.								x
Glechoma hederacea L.								x
Lamium album L.								x
Ranunculus repens L.								x
Reseda luteola L.								x
Taraxacum sect. Ruderalia Wiggers								x
Veronica persica Poir.								x
Poaceae								
Arrhenatherum elatius (L.) P. Beauv.	x	x	x	x	x	x	x	x
Dactylis glomerata L.	x	x	x	x	x	x	x	x
Schedonorus arundinaceus Schreb.	x	x	x	x	x	x	x	x

Supplementary methods: Functional characterization of the plant communities

Flower traits were measured on the plants observed in the experimental field or on plants found in the surroundings for the species that did not emerge (to compare the sown and realized functional diversities). Between 2015 and 2017, we recorded the species flowering (i.e. for which at least 20% of the individuals had flowers) every week. We then determined two traits: the calendar **week number at which flowering began** and the **duration of flowering** (number of weeks), averaged over the three years of field observations. We also checked the entire plant for the **presence of extrafloral nectar** (0 = none, 1 = present).

Flower traits were measured at peak flowering, on a minimum of five or ten flowers collected from different plants, for spontaneous and sown species, respectively. Flowers were sampled early in the morning and placed in water for at least one hour before observations. Small flowers were examined under a binocular microscope (Leica M80, 60×) linked to a video camera (Moticam 10, Motic).

As a proxy for flower attractiveness (Fiedler and Landis, 2007), **flower diameter** (maximum flower size at the tip of the petals) **or inflorescence diameter** (for grouped flowers) was measured under a binocular microscope or with a ruler (for flowers > 20 mm in diameter).

The **amount of floral nectar** was assessed in a qualitative manner on a three-point scale (0 = none, 1 = a little, 2 = plenty). We assessed the accessibility of the nectar to parasitoids (as described in Appendix 4), by measuring four specific flower traits:

- Flower **opening diameter** or the width of the narrowest constraint limiting the access to the flower. Opening diameter was generally measured at the extremity of unfused petals or at the point of fusion of the petals (or tepals or sepals if no petals were present), as appropriate. Alternatively, we measured the narrowest constraint limiting access to the flower. For example, the structures measured included fringes of dense hairs (*Lamium album*, *Malva sylvestris*) or the extremity of the petals of closed flowers (many members of the Fabaceae*, Fumaria officinalis*). In each case, we measured the narrowest zone, in which the insect head might become blocked. FGor petals with inward corolla protuberances restricting corolla width (e.g. *Myosotis arvensis*), we measured the diameter of the opening at this location.

- **Corolla height**, measured from the corolla opening to the base of the perianth

- **Nectar depth** is the distance between the corolla opening (or the narrowest constraint) and the top of the zone in which nectar is found

- **Nectar tube diameter** at the top of the zone in which nectar is present.

We also extracted measurements for other traits from previous studies. We assessed the attractiveness of flowers, using data for basic **flower colour** and the presence of a **UV reflection pattern** extracted from the BiolFlor database (Kühn et al. 2004). Missing data were replaced with our own observations (for colour) or with data from the Floral Reflectance Database (Arnold et al. 2010). As a proxy for the provision of habitats to insects, we also collected trait data for **leaf distribution** (rosette, semi-rosette or leaves distributed along the stem), **vegetative** and **flowering heights** from the LEDA database (Kleyer *et al.*, 2008).

We then calculated the functional diversity of the plant assemblages on the basis of the following traits: flower or inflorescence diameter, amount of floral nectar, nectar depth, presence of extrafloral nectar, date on which flowering began, duration of flowering, flower colour, presence of a UV reflection pattern, vegetative and flowering heights and leaf distribution. Here, we use only nectar depth as a proxy for nectar accessibility, to avoid collinearity between flower traits.

We calculated (1) functional dispersion, defined as the abundance-weighted mean distance of individual species from the centroid of all species in the trait space (Laliberté and Legendre, 2010) and (2) Rao quadratic entropy, *i.e.* the abundance-weighted sum of pairwise functional distances between species (Rao 1982). Functional dispersion and Rao quadratic entropy were strongly correlated (marginal $R^2 = 0.95$, $p < 10^{-4}$, n=168, result obtained with a mixed model with treatment number as a random effect). We therefore considered only functional dispersion, which best reflects niche diversity, as a measurement of functional diversity. These metrics were calculated with the FD package (Laliberté *et al.*, 2014).

Supplementary Table S3. List of the traits involved in plant-parasitoid interactions taken into account in this study and associated hypotheses based on Gardarin *et al.* (2018).

Mechanisms	Plant traits	Parasitoid traits
**Provision of physical habitats**		
Plants provide physical habitats for all life stages, and can modify the microclimate. The structural properties of non-crop vegetation, such as density, height and litter quantity, are known to influ- ence the distribution of soil-dwelling arthropods (Griffiths et al. 2008). Plant structural traits such as growth form, height, architecture (density and orientation of branches and leaves, leaf morphology) shape arthropod habitats (Parolin et al. 2012).	Leaf distribution<br>Vegetative height<br>Flowering height	
**Ressource provision**		
The carbohydrate resources provided by plants increase the longevity, reproduction and dispersal of their consumers (Wäckers et al. 2005).. Plants may act as occasional and supplementary food sources, but the resources they supply may also be of crucial importance, in synovigenic species of parasitoids for instance (Jervis et al. 2004), with nectar and pollen proteins having strong effects on adult performance and egg maturation. Extrafloral nectar generally has a higher sugar concentration, whereas honeydew has a lower nutritional quality than nectar (Lee et al. 2004).	Presence of floral nectar<br>Presence of extrafloral nectar<br>Amount of floral nectar	
**Flower or inflorescence attractiveness**		
Olfactory, gustatory and visual signals facilitate the recognition and detection of resources, but may also be repellent, depending on the preferences of arthropod groups. Visual signals, such as plant height, flower height, inflorescence size and colour, are involved in resource detection, and a high degree of visual attractiveness increases the abundance of natural enemies (Fiedler and Landis 2007).	Diameter<br>Color<br>UV reflectance pattern	
**Temporal availability of nectar**		
The synchrony between the plant and arthropod cycles determines the likelihood of interaction (Welch and Harwood 2014). The seasonal availability of resources depends on plant phenology. The phenological match between flowering period and arthropod floral resource requirements is crucial for completion of the life cycles of both herbivores and their natural enemies.	Date of flowering onset<br>Duration of flowering	Period of adult activity
**Floral nectar accessibility**		
In flower-visiting arthropods, head or body size may physically restrict access to floral resources in flowers with a small corolla diameter. In such cases, the plant–arthropod interaction depends on the length of the mouthparts. A correlation between nectar holder depth and the proboscis length of the flower visitor has been observed in several insect groups, especially in pollinators. Short corolla flowers favour hoverflies, whereas bumblebees prefer long corollas (Campbell et al. 2012). As a result, flower size is one of the most important variables determining the abundance and diversity of flower visitors and their size (Ibanez 2012; Stang et al. 2006; van Rijn and Wäckers 2016). By contrast, extrafloral nectar is generally produced on exposed nectaries, with no size constraints on its accessibility.	Flower opening diameter<br>Corolla height<br>Nectar depth<br>Nectar tube diameter	Proboscis length<br>Proboscis width<br>Head width

References

Arnold, S.E.J., Faruq, S., Savolainen, V., McOwan, P.W. & Chittka, L. (2010) FReD: The Floral Reflectance Database — A web portal for analyses of flower colour. *PLoS One,* **5,** e14287.

Fiedler, A.K. & Landis, D.A. (2007) Plant characteristics associated with natural enemy abundance at Michigan native plants. Environmental Entomology, 36, 878-886.

Gardarin A, Plantegenest M, Bischoff A, Valantin-Morison M (2018) Understanding plant-arthropod interactions in multitrophic communities to improve conservation biological control: useful traits and metrics. J Pest Sci 91 (3):943-955. doi:10.1007/s10340-018-0958-0

Kleyer, M., Bekker, R.M., Knevel, I.C., Bakker, J.P., Thompson, K., Sonnenschein, M., Poschlod, P., Van Groenendael, J.M., Klimeš, L., Klimešová, J. *et al.* (2008) The LEDA Traitbase: a database of life-history traits of the Northwest European flora. *Journal of Ecology,* **96,** 1266-1274.

Kühn, I., Durka, W. & Klotz, S. (2004) BiolFlor - a new plant-trait database as a tool for plant invasion ecology. *Diversity and Distributions,* **10,** 363-365.

Laliberté, E. & Legendre, P. (2010) A distance-based framework for measuring functional diversity from multiple traits. *Ecology,* **91,** 299-305.

Laliberté, E., Legendre, P. & Shipley, B. (2014) FD: measuring functional diversity from multiple traits, and other tools for functional ecology. R package version 1.0-12.

Rao, C.R. (1982) Diversity and dissimilarity coefficients - A unified approach. *Theoretical Population Biology,* **21,** 24-43.

Supplementary methods: Assessment of the parasitism of herbivorous pests of faba bean and oilseed rape

• Parasitism of larvae of *Bruchus rufimanus* (Bohemann, 1833) (Coleoptera: Chrysomelidae)
At the end of July 2016, when the faba beans were fully mature, we collected 100 pods, each containing two to three seeds, from different plants located at the specified distances from each wildflower strip. The pods were placed in plastic milk bottles closed with an insect proof net, and were stored at room temperature (about 20°C) until November 2016. We then examined the seeds, and recorded the large exit holes of *B. rufimanus* and the small exit holes made by their parasitoids (Camillo-Perdromo et al., 2019). If signs of a hole were observed but the insect had not emerged from the seed, we enlarged the hole with a needle to determine whether it contained an adult *B. rufimanus* or a parasitoid. The parasitoids were identified as *Triaspis sp.* and belonged probably to the *T. thoracica complex* (Curtis, 1860) (Hymenoptera: Braconidae) parasitizing of several *Bruchus* species (de Luca *et al.*, 1965). The parasitism rate was calculated as the number of parasitoid holes divided by the total number of holes (parasitoid and *B. rufimanus* holes) observed in the seed sample.

• Parasitism of larvae of *Ceutorhynchus pallidactylus* (Panzer, 1795) (Coleoptera, Curculionidae) and *Psylliodes chrysocephala* (Linnaeus, 1758) (Coleoptera: Chrysomelidae)
In April 2017, we collected 100 (when possible) last instar larvae of each species from the petioles of basal leaves of oilseed rape. The collection of larvae was very time-consuming, and we were, therefore unable to sample these larvae at a distance of 20 m for all assemblages (only the LFMS2, HFMS2, HFHS1, HFHS2 and control treatments were sampled at this distance). The larvae were placed in hermetic plastic boxes (9.5 cm in diameter, 7 cm high) two-thirds filled with sieved dry soil. The *C. pallidactylus* and for *P. chrysocephala* larvae were placed in separate boxes. The rearing boxes were placed at room temperature (about 20°C) for six months, then at 4°C for five months to simulate winter temperatures, before being returned to room temperature. We recorded the emerging adults of *P. chrysocephala* and *C. pallidactylus* (which developed from non-parasitized larvae) during the first two months after collection in the field. Parasitoids emerged after the release from low temperatures and were recorded over a period of two months, until no further emergences occurred. For both hosts, the parasitoids were morphologically similar to *Tersilochus microgaster* (Szépligeti, 1899) (Hymenoptera, Ichneumonidae) and *T. obscurator* (Aubert, 1959) (Hymenoptera, Ichneumonidae), according

to published identification tools (Vidal, 2007; Barrari *et al.,* 2005; Robert *et al.*, 2019), and could not be assigned to either species with certainty. They are described as being specific of theirs hosts among oilseed rape pests (Ulber *et al*., 2010).

Finally, we searched the soil in the rearing boxes for any remaining cocoons of parasitoids or non-parasitized adults of the host species that did not emerge and died after metamorphosis because of non-optimal rearing conditions. Parasitism rates for *C. pallidactylus* and *P. chrysocephala* were then calculated as the number of adult parasitoids that had emerged or were recovered from the soil divided by the sum of parasitoids and adult hosts that had emerged or were recovered from the soil.

• Parasitism of larvae of *Brassicogethes aeneus* (Fabricius, 1775) (Coleoptera, Nitidulidae)
In early May 2017, we collected 100 (when possible) last instar larvae from oilseed rape flowers, which we stored in 70° alcohol. Under a binocular microscope, we recorded the presence of *Tersilochus heterocerus* (Thomson, 1889) (Hymenoptera: Ichneumonidae) eggs in the larvae, this species being the main parasitoid of *B. aeneus* in the area (Rusch *et al.*, 2011). Parasitism rates were calculated as the number of larvae containing at least one parasitoid egg divided by the total number of hosts.

• Parasitism of larvae of *Dasineura brassicae* (Winnertz, 1853) (Diptera: Cecidomyiidae)
Immediately after the oilseed rape harvest in July 2017, we collected soil samples with a spade, from an area of 20 cm × 20 cm and a depth of 5 cm. The soil samples were stored for two months at 5°C. They were then gently washed in a sieve with a 1-mm mesh, to remove all the fine soil particles. For each sample, a minimum of 150 white cocoons of *Dasineura brassicae* were recovered from the particles remaining in the sieve. We dissected the cocoons under a binocular microscope, and recorded the numbers of cocoons containing non-parasitized host nymphs or adult micro-hymenoptera (from one to three individuals).
Three morpho-species of micro-hymenoptera were retrieved:
- 15.8% of individuals belonged to *Inostemma sp*. (Hymenoptera: Platygastridae), identified as described by Delvare (2010) and specialized on *D. brassicae* among oilseed rape pests (Ulber *et al.*, 2010).
- 71.9% of individuals belonged to morpho-species 1
- 12.3% of individuals belonged to morpho-species 2.

We assumed that all were parasitoids, although we cannot exclude that morpho-species were

hyper-parasitoids. Empty cocoons were not taken into account. The parasitism rate was calculated as the number of cocoons containing at least one micro-hymenoptera divided by the total number of non-empty cocoons.

References

Barari, H., Ferguson, A.W., Piper, R.W., Smith, E., Quicke, D.L.J. & Williams, I.H. (2005) The separation of two hymenopteran parasitoids, *Tersilochus obscurator* and *Tersilochus microgaster* (Ichneumonidae), of stem-mining pests of winter oilseed rape using DNA, morphometric and ecological data. *Bulletin of Entomological Research,* **95,** 299-307.

de Luca Y (1965) Catalogue des métazoaires parasites et prédateurs Bruchides (Coleoptera). *J Stored Prod Res* 1:51-98

Delvare, G. (2010) Reconnaissance des auxiliaires (hyménoptères parasitoïdes) en vue de leur utilisation en vue de leur utilisation en lutte biologique et intégrée, 166 p.

Robert, C., Bothorel, S., Luce, S., Lauvernay, A., Leflon, M., Delvare, G., Streito, J.C., Pierre, E., Cruaud, P., Ollivier, M. *et al.* (2019) COLEOTOOL - Développement d'outils moléculaires en vue d'identifier les principaux charançons ravageurs du colza et leurs auxiliaires parasitoïdes. *Innovations agronomiques,* **71,** 181-200.

Rusch, A., Valantin-Morison, M., Sarthou, J.P. & Roger-Estrade, J. (2011) Multi-scale effects of landscape complexity and crop management on pollen beetle parasitism rate. *Landscape Ecology,* **26,** 473-486.

Vidal, S. (2007) Identification of hymenopterous parasitoids associated with oilseed rape pests. *Biocontrol of oilseed rape pests* (ed. D.V. Alford), pp. 161-180. Blackwell, Oxford.

Supplementary methods: Estimation of nectar accessibility to parasitoids

The accessibility of floral nectar depends on the morphological match between insects and flowers. We developed a geometric model, adapted from that described by van Rijn and Wäckers (2016), and parameterized with four flower traits and three insect traits (Figure S1).

The flower traits included are generally measured on the corolla, but sometimes on the perianth, in situations in which the sepals and petals have similar functions:

- *w* is generally the width of the flower opening. It was generally measured at the extremity of unfused petals or at the point of petal (or tepal or sepal if no petals) fusion, as appropriate. However, *w* could also be the width of the narrowest constraint limiting access to the flower. For example, it could be fringes of dense hairs (*Lamium album*, *Malva sylvestris*) or the extremity of the petals of closed flowers (many members of the Fabaceae, *Fumaria officinalis*). We systematically recorded the width of the narrowest zone, in which the head of the insect might be blocked. For flowers with petals displaying inward corolla protuberances restricting corolla width (e.g. *Myosotis arvensis*), we measured the diameter of the opening at this location.
- *h* is corolla height, measured from the opening of the corolla to the base of the perianth
- *p* is nectar depth, measured as the distance between the opening of the corolla (or the narrowest constraint) and the upper part of the zone containing nectar
- *d* is the nectar tube diameter in the upper part of the zone containing nectar.

Three insect traits (Table S3) were measured for each parasitoid species or morphospecies:

- *r*, the radius of the insect head (half the diameter, assuming a spherical head)
- *z*, proboscis length
- *x*, proboscis width.

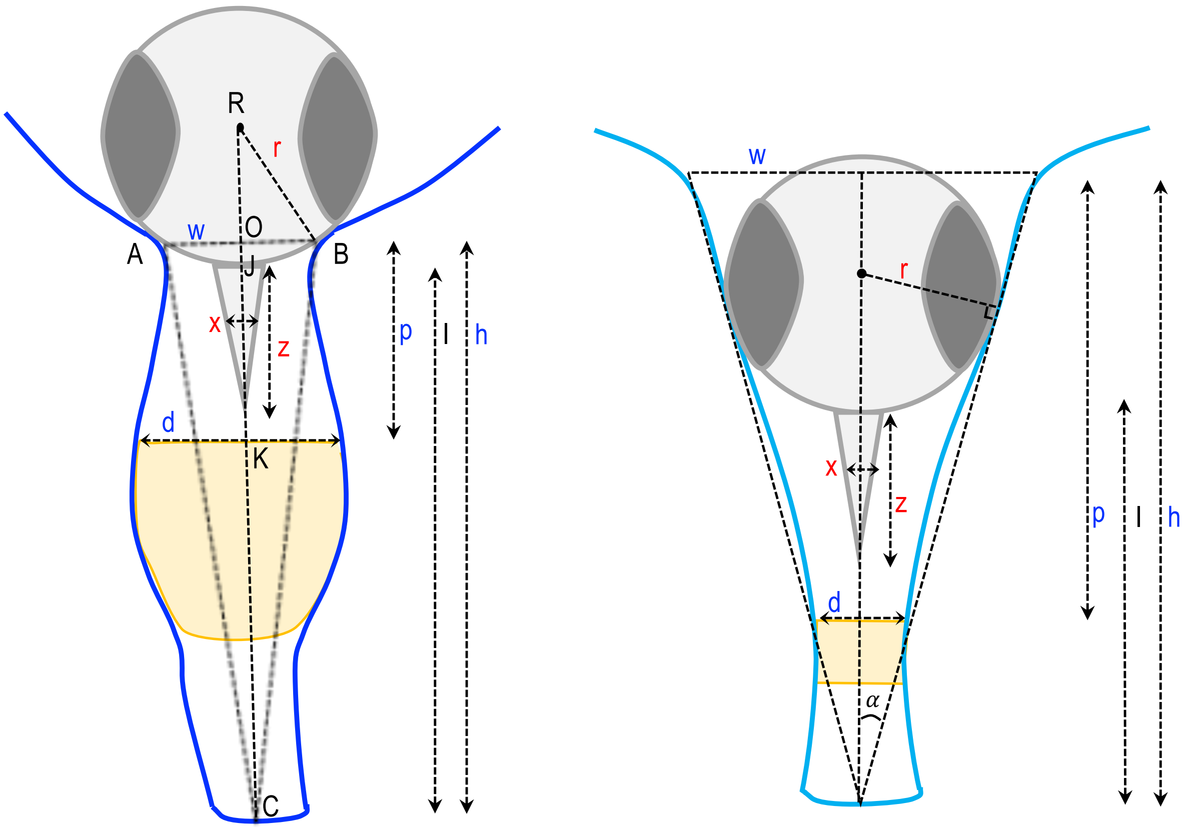

Supplementary Figure S1. Schematic diagram of the geometric constraints determining the accessibility of floral nectar to insects. The location of the nectar is shown in yellow. We distinguished the following situations: (1, left) when insect head remains outside the corolla due to size constraints and (2, right) when the head penetrates inside the corolla. The measured insect traits (*r, x, z*) are indicated in red, and the measured flower traits (*w, h, p, d*) are indicated in blue. The distance *l* between the extremity of the head and the base of the corolla was calculated to determine nectar accessibility. See the text for the meaning of the symbols. This figure was created using Microsoft Powerpoint version 16.16.14 (https://www.microsoft.com).

Supplementary Table S4. Morphological measurements of the heads and mouthparts of the parasitoids

Host	Parasitoid	Period of parasitoid activity	Head diameter (mm)	Proboscis length (mm)	Proboscis width (mm)
Bruchus rufimanus (Bohemann, 1833)	*Triaspis thoracica* (Curtis, 1860)	May (Ward, 2018)	0.828 ± 0.038	0	0
Psylliodes chrysocephala (Linnaeus, 1758)	*Tersilochus sp.* 1 (Hymenoptera, Ichneumonidae)	Mid-February to end of March (Ulber *et al.*, 2010; personal field observations)	0.834 ± 0.058	0.168 ± 0.029	0.047 ± 0.016
Ceutorhynchus pallidactylus (Panzer, 1795)	*Tersilochus sp.* 2 (Hymenoptera, Ichneumonidae)	March and April (Ulber *et al.*, 2010)	0.866 ± 0.062	0.197 ± 0.045	0.032 ± 0.007
Brassicogethes aeneus (Fabricius, 1775)	*Tersilochus heterocerus* (Thomson, 1889)	April (Ulber *et al.*, 2010; personal field observations)	0.606 ± 0.040	0.148 ± 0.028	0.028 ± 0.012
Dasineura brassicae (Winnertz, 1853)	Inostemma sp. (Hymenoptera: Platygrastridae) — 15.8% of individuals	May and June (Ulber *et al.*, 2010)	0.310 ± 0.108	0.023 ± 0.002	0.052 ± 0.026
	Morpho-species 1 — 71.9% of individuals	May and June (Ulber *et al.*, 2010)	0.335 ± 0.008	0.095 ± 0.017	0.012 ± 0.001
	Morpho-species 2 — 12.3% of individuals	May and June (Ulber *et al.*, 2010)	0.325 ± 0.038	0.070 ± 0.006	0.012 ± 0.001

The constraints limiting nectar accessibility are summarized on a decision tree (Figure 4). In particular, we needed to calculate the distance between the parasitoid head and the site at which the nectar was found. We distinguished two cases, according to the ability of the insect to penetrate the flower.

• **<u>Case 1</u>**: the parasitoid has a head too large to enter the corolla ($2r \geq w$). In this case, we calculated the distance l between the extremity of the head and the base of the corolla: l = JC = h – OJ.

h, the total corolla height, is a measured trait. We needed to calculate the distance OJ.

We applied Pythagoras' theorem to the RBO triangle: $r^2 = OR^2 + OB^2$, so $OR = \sqrt{r^2 - OB^2}$.

Given that RJ = r = OR + OJ, we obtain: $OJ = r - \sqrt{r^2 - \left(\frac{W}{2}\right)^2}$.

Given that l = h – OJ, we obtain $l = h - (r - \sqrt{r^2 - \left(\frac{W}{2}\right)^2})$.

The nectar is accessible when z, the length of the proboscis, is greater than or equal to the distance JK between the parasitoid head and the upper part of the nectar-containing zone.

We have: l = JK + (h – p), where p is nectar depth, measured from the top of the corolla (or constraint).

And finally: $z \geq$ JK therefore implies that $z \geq l - h + p$.

• **Case 2**: the head can penetrate the corolla ($2r < w$). We needed to calculate the distance l between the extremity of the head and the base of the corolla.

First, $\sin(\alpha) = r/(r + l)$, so $l = r\frac{1-\sin(\alpha)}{\sin(\alpha)}$.

In addition, $\tan(\alpha) = \frac{w/2}{h}$, so $\alpha = \arctan\left(\frac{w}{2h}\right)$.

Thus: $l = r\frac{1-\sin\left(\arctan\left(\frac{w}{2h}\right)\right)}{\sin\left(\arctan\left(\frac{w}{2h}\right)\right)}$.

As in case 1, the nectar is accessible when z, the length of the proboscis, is greater than or equal to the distance between the parasitoid head and the upper limit of the nectar-containing zone, *i.e.* when $z \geq l - h + p$.

References

Ulber, B., Williams, I.H., Klukowski, Z., Luik, A. & Nilsson, C. (2010) Parasitoids of oilseed rape pests in Europe: key species for conservation biocontrol. *Biocontrol-based integrated management of oilseed rape pests* (ed. I.H. Williams), pp. 45-76. Springer, Netherlands.

van Rijn, P.C.J. & Wäckers, F.L. (2016) Nectar accessibility determines fitness, flower choice and abundance of hoverflies that provide natural pest control. *Journal of Applied Ecology,* **53,** 925-933.

Ward, R.L. (2018) The biology and ecology of *Bruchus rufimanus* (bean seed beetle). Ph.D. thesis, Newcastle University, United-Kingdom.

Supplementary results

Assessment of the populations of crop herbivores

In oilseed rape, we characterized the pressure of the adult herbivore populations colonizing the field and at the origin of the new generation, in which we measured the larval parasitism. The damage is caused by the adults of this generation (for B. aeneus) or by the larvae of the following generation (all other studied herbivores).

Five yellow pan traps (28 cm diameter, filled with water and a drop a scentless detergent) were placed in the 6.5 ha-experimental field, with one trap at 20 m from each field corner and one trap at the centre of the field. The pan traps were monitored weekly during the whole oilseed rape cycle in 2016-17.

In *P. chrysocephala*, there were 265 ± 70 adults per trap captured from September to November on the experimental field, which is substantially higher than in conventional fields in the region (45 adults over a network of 41 fields monitored by agricultural extension services, https://driaaf.ile-de-france.agriculture.gouv.fr/Epidemiosurveillance-et-Bulletin).

Concerning *B. aeneus*, we captured 1072 ± 408 adults on average per trap in March and in April, which is clearly higher than what can be observed in the literature. In Hiiesaar *et al.* (2003), there were about 180 adults on average per trap during the equivalent period, and in Hatt *et al.* (2015), there were about 60 adults on average per trap during the equivalent period).

Concerning *C. pallidactylus*, we captured 35 ± 8 adults in March and April. As this insect is poorly studied and monitored, we do not have reference values with comparable methods to make comparisons. However, Hatt *et al.* (2015) captured about 50 *Ceutorrhynchus spp*. (several undistinguished species) per trap during an equivalent period.

We did monitor the abundance of *D. brassicae* adults nor larvae for which yellow pan traps are not adapted.

Concerning faba bean, we measured seed damage resulting for the herbivory of *B. rufimanus* on all samples taken in each experimental treatment (each plant assemblage × 2 distances). On average, 62 (± 13) % of the seeds were damaged. This is relatively high, but it falls within the range of the values we observe in France, in a network of farmer's fields followed since several years (25% of the seeds are damaged on average, but with high variations between fields, unpublished personal observations).

References

Hatt, S., Uyttenbroeck, R., Lopes, T.M., Paul, A., Danthine, S., Bodson, B., Piqueray, J., Monty, A., Francis, F., 2015. Do wildflower strips favor insect pest populations at field margins? Agriculture and agricultural science procedia. 6, 30-37.

Hiiesaar, K., Metspalu, L., Lääniste, P., Jõgar, K., Kuusik, A., 2003. Insect pests on winter oilseed rape studied by different catching methods. 1.

Supplementary results: tables

Supplementary Table S5. Effects of the type of assemblage on (1) the parasitism of five insect herbivore pests of faba bean and oilseed rape, and (2) multi-species parasitism, measured at 5 and 20 m from the flower strip. Multi-species parasitism, quantified with a multi-threshold approach, was assessed as the number of herbivorous species for which the parasitism rate exceeded a given percentage (thresholds from 10 to 90%) of the maximum parasitism achieved for each species. Generalised linear mixed effect models were used, assuming a binomial (parasitism rates) or Poisson (multi-species parasitism) error distribution, and including the strip as a random effect. All explanatory variables were scaled. We report the degrees of freedom for explanatory variables (df), the residual degrees of freedom of the model (df resid) and the marginal and conditional coefficients of determination (R^2m and R^2c).

Species	Variables	df, df resid	χ^2	P (>χ^2)	R^2 m	R^2 c	AIC
Bruchus rufimanus	Assemblage	8, 35	59.732	**< 10^{-4}**	0.81	0.83	461.3
	Distance	1, 35	18.311	**< 10^{-4}**			
	Assemb. × Dist.	8, 35	5.9695	0.6506			
Psylliodes chrysocephala	Assemblage	8, 25	12.835	0.1177	0.50	0.49	144.4
	Distance	1, 25	2.6286	0.1050			
	Assemb. × Dist.	4, 25	5.6815	0.2242			
Ceurorrhynchus pallidactylus	Assemblage	8, 25	17.834	**0.0225**	0.48	0.46	206.9
	Distance	1, 25	2.5598	0.1096			
	Assemb. × Dist.	4, 25	5.1832	0.2690			
Brassicogethes aeneus	Assemblage	8, 35	6.9641	0.5405	0.57	0.44	312.8
	Distance	1, 35	0.2419	0.6228			
	Assemb. × Dist.	8, 35	23.675	**0.0026**			
Dasineura brassicae	Assemblage	8, 35	16.2708	**0.03867**	0.46	0.44	456.4
	Distance	1, 35	0.5827	0.44524			
	Assemb. × Dist.	8, 35	12.6361	0.1250			
Multispecies parasitism	Assemblage	8, 334	10.815	0.2124	0.52	0.53	1179.6
	Distance	1, 334	1.8695	0.1715			
	Thresholds	1, 334	215.11	**< 10^{-4}**			
	Assemb. × Dist.	4, 334	4.9470	0.2928			
	Ass.×Thresholds	8, 334	23.678	**0.0026**			
	Dist.×Thresholds	1, 334	1.7015	0.1921			

Supplementary Table S6. Effects of the type of assemblage on (1) the parasitism of five insect herbivore pests of faba bean and oilseed rape, and (2) multi-species parasitism, tested for each distance separately. See legend of Table S5 for more details.

Species	Distance	Variables	df, df resid	χ^2	P (>χ^2)	R²m	R²c	AIC
Bruchus rufimanus	5 m	Assemblage	8, 17	34.039	**< 10^{-4}**	0.72	0.75	209.0
	20 m	Assemblage	8, 17	32.140	**< 10^{-4}**	0.71	0.75	252.5
Psylliodes chrysocephala	5 m	Assemblage	8, 17	8.082	0.43	0.28	0.32	100.5
	20 m	Assemblage	4, 7	10.203	**0.04**	0.74	0.75	44.2
Ceutorrhynchus pallidactylus	5 m	Assemblage	8, 17	20.249	**0.0094**	0.56	0.57	139.0
	20 m	Assemblage	4, 7	2.33	0.68	0.16	0.16	71.7
Brassicogethes aeneus	5 m	Assemblage	8, 17	20.057	**0.0101**	0.54	0.56	155.7
	20 m	Assemblage	8, 17	10.659	0.22	0.34	0.55	161.0
Dasineura brassicae	5 m	Assemblage	8, 17	12.259	0.14	0.39	0.54	201.2
	20 m	Assemblage	8, 17	15.498	0.05	0.46	0.46	253.5
Multi-species parasitism	5 m	Thresholds	1, 223	5.884	**< 10^{-4}**	0.53	0.53	802.3
		Assemblage	8, 223	146.609	0.66			
		Ass.×thresholds	8, 223	15.38	0.05			
	20 m	Thresholds	1, 105	67.038	**< 10^{-4}**	0.54	0.54	388.2
		Assemblage	4, 105	9.156	0.05			
		Ass.×thresholds	4, 105	8.044	0.09			

Supplementary Table S7. Result of the morphological trait-matching decision tree determining the ability of parasitoids to feed on nectar. The interaction was found possible (1) or not possible (0). It was not evaluated (ne) for species not flowering during the period of adult parasitoid activity. Species that did not flower or produce nectar during any period of adult parasitoid activity are not shown.

Parasitoid species	**Tersilochus sp. 1 (host: *P. chrysocephala*)**	***Tersilochus sp.* 2 (host: *C. pallidactylus*)**	***Tersilochus heterocerus* (host: *B. aeneus*)**	***Triaspis thoracica* (host: *B. rufimanus*)**	***Inostemma sp.* (host: *D. brassicae*)**	**Morpho-species 1 (host: *D. brassicae*)**	**Morpho-species 2 (host: *D. brassicae*)**
Total number of species	78	78	78	68	67	78	78
Flowering species	16	16	23	51	59	59	59
Species producing nectar	16	16	22	44	49	49	49
Species with accessible nectar	13	13	17	20	36	36	36
Achillea millefolium	ne	ne	ne	0	1	1	1
Ajuga reptans	ne	ne	1	ne	ne	ne	ne
Alliaria petiolata	1	1	1	ne	1	1	1
Anthemis tinctoria	ne	ne	ne	0	1	1	1
Anthriscus caucalis	ne	ne	1	ne	1	1	1
Anthriscus sylvestris	ne	ne	1	ne	1	1	1
Barbarea vulgaris	1	1	1	1	1	1	1
Bellis perennis	0	0	1	1	1	1	1
Capsella bursa-pastoris	1	1	1	1	1	1	1
Cardamine hirsuta	1	1	1	ne	1	1	1
Centaurea cyanus	ne	ne	ne	1	1	1	1
Centaurea scabiosa	ne	ne	ne	ne	1	1	1
Cynoglossum officinale	ne	ne	0	0	1	1	1
Echium vulgare	ne	ne	ne	1	1	1	1
Epilobium tetragonum	ne	ne	ne	0	0	0	0
Euphorbia cyparissias	1	1	1	ne	ne	ne	ne
Euphorbia helioscopia	1	1	1	1	1	1	1
Fallopia convolvulus	ne	ne	ne	ne	1	1	1
Galium aparine	ne	ne	1	1	1	1	1
Galium mollugo	ne	ne	ne	1	1	1	1
Geranium dissectum	ne	ne	1	1	1	1	1
Geranium molle	ne	ne	1	1	1	1	1
Geum urbanum	ne	ne	1	1	1	1	1
Glechoma hederacea	1	1	1	ne	ne	ne	ne
Heracleum sphondylium	ne	ne	1	1	1	1	1
Hesperis matronalis	ne	ne	0	1	1	1	1
Hypochaeris radicata	ne	ne	ne	0	1	1	1
Lamium hybridum	1	1	1	ne	ne	ne	ne
Lamium purpureum	1	1	1	ne	ne	ne	ne
Lapsana communis	ne	ne	ne	0	0	0	0
Leucanthemum vulgare	ne	ne	1	1	1	1	1
Lotus corniculatus	ne	ne	ne	0	0	0	0
Malva sylvestris	ne	ne	ne	0	0	0	0
Matricaria perforata	ne	ne	ne	0	1	1	1
Medicago lupulina	ne	ne	ne	0	0	0	0
Medicago sativa	ne	ne	ne	0	0	0	0
Melilotus officinalis	ne	ne	ne	0	0	0	0
Myosotis arvensis	0	0	0	0	1	1	1
Onobrychis viciifolia	ne	ne	ne	0	0	0	0
Pastinaca sativa	ne	ne	1	1	1	1	1
Picris echioides	ne	ne	ne	1	1	1	1
Picris hieracioides	ne	ne	ne	0	1	1	1
Polygonum aviculare	ne	ne	ne	ne	1	1	1
Securigera varia	ne	ne	ne	0	0	0	0
Senecio vulgaris	0	0	0	0	1	1	1
Silene dioica	1	1	1	1	1	1	1
Sisymbrium officinale	ne	ne	ne	1	1	1	1
Sonchus asper	ne	ne	1	1	1	1	1
Stellaria media	1	1	1	1	1	1	1
Taraxacum officinale	1	1	1	ne	ne	ne	ne
Torilis arvensis	ne	ne	ne	ne	1	1	1
Trifolium pratense	ne	ne	ne	0	0	0	0
Trifolium repens	ne	ne	ne	1	1	1	1
Valerianella sp.	ne	ne	ne	0	0	0	0
Veronica hederifolia	1	1	1	ne	ne	ne	ne
Veronica persica	1	1	1	1	ne	ne	ne
Veronica polita	1	1	1	ne	ne	ne	ne
Vicia sativa	1	1	1	ne	ne	ne	ne

Supplementary Table S8. Effects of the composition and structure (percentage plant cover providing accessible nectar, species richness and functional dispersion) of the flower strip plant communities on rates of parasitism in five herbivorous insect pests and on global multi-species parasitism (quantified by a multi-threshold approach). We show here the best models (ΔAIC from the best model < 2) obtained with the multi-model inference procedure comparing all possible combinations of the fixed effect variables (linear and quadratic terms) and their interactions. Generalized linear mixed effect models were used, assuming a binomial (parasitism rates) or Poisson (multi-species parasitism) error distribution, and including the strip as a random effect. All explanatory variables were scaled.

Parasitism at 5 m from the strip

Bruchus rufimanus	Models with ΔAIC < 2							
**Explanatory fixed variables**	1	2	3	4	5	6	7	8
Intercept	-0.816	-0.8269	-0.8115	-0.8195	-0.8453	-0.8137	-0.8131	-0.8235
Functional dispersion^2	-0.084	-0.070	-0.088		-0.077	-0.064	-0.087	-0.085
Functional dispersion	-0.056				-0.082		-0.065	-0.042
Nectar resources^2					0.024			0.001
Nectar resources	0.101	0.079	0.082	0.13	0.060	0.109	0.096	0.092
Species number^2								-0.002
Species number			-0.044				-0.016	
Func. disp. × Nectar res.				-0.127		-0.035		
AIC	199.0	199.1	199.4	200.1	200.2	200.9	200.9	200.9
ΔAIC from best model	0.00	0.18	0.40	1.17	1.26	1.84	1.90	1.92
Marginal R^2	0.70	0.70	0.72	0.71	0.71	0.72	0.72	0.72
Conditional R^2	0.75	0.73	0.74	0.74	0.74	0.75	0.75	0.75

Psylliodes chrysocephala	Models with ΔAIC < 2							
**Explanatory fixed variables**	1	2	3	4	5	6	7	8
Intercept	-2.415	-2.426	-2.415	-2.334	-2.498	-2.309	-2.420	-2.444
Functional dispersion^2			-0.268		-0.115			-0.291
Functional dispersion		-0.194						
Nectar resources^2			0.271		0.096			0.288
Nectar resources	0.373	0.453		0.357	0.297	0.398	0.412	
Species number^2						-0.115		
Species number							-0.089	0.183
AIC	88.6	89.7	89.7	90.1	90.1	90.3	90.4	90.4
ΔAIC from best model	0.00	1.09	1.12	1.46	1.49	1.72	1.74	1.84
Marginal R^2	0.21	0.24	0.24	0.23	0.23	0.22	0.22	0.27
Conditional R^2	0.25	0.28	0.27	0.27	0.26	0.26	0.26	0.31

Parasitism at 20 m from the strip

Bruchus rufimanus	Models with ΔAIC < 2									
**Explanatory fixed**	1	2	3	4	5	6	7	8	9	10
Intercept	-0.658	-0.663	-0.769	-0.738	-0.769	-0.755	-0.678	-0.680	-0.674	-0.686
Functional dispersion^2	-0.105	-0.147		-0.079	-0.085	-0.085	-0.107	-0.090	-0.149	-0.126
Functional dispersion			-0.111	-0.091				-0.072		
Nectar resources^2			0.059	0.053	0.078		0.024		0.025	0.092
Nectar resources					-0.071					-0.079
Species number^2		0.047							0.048	
Species number			0.0162	0.134	0.097	0.040		0.088		0.012
Func. disp. × Sp. number			-0.070							
AIC	225.6	225.8	226.2	226.2	226.3	226.8	226.9	227.0	227.2	227.2
ΔAIC from best model	0.00	0.26	0.61	0.61	0.78	1.28	1.39	1.40	1.59	1.66
Marginal R^2	0.85	0.85	0.85	0.85	0.85	0.85	0.85	0.85	0.85	0.85
Conditional R^2	0.87	0.87	0.87	0.87	0.88	0.87	0.87	0.87	0.87	0.88

Psylliodes chrysocephala	Models with ΔAIC < 2			
**Explanatory fixed**	1	2	3	4
Intercept	-2.136	-2.085	-2.126	-2.131
Functional dispersion				-0.073
Nectar resources		-0.253		
Species number			-0.084	
AIC	52.9	53.8	54.6	54.6
ΔAIC from best model	0.00	1.10	1.85	1.87
Marginal R^2	0	0.06	0.01	0.01
Conditional R^2	0.11	0.17	0.12	0.12

Table S8 (continued).

Parasitism at 5 m from the strip

Ceutorhynchus pallidactylus	**Models with ΔAIC < 2**				
**Explanatory fixed variables**	1	2	3	4	5
Intercept	-1.264	-1.264	-1.288	-1.264	-1.256
Functional dispersion²					-0.011
Nectar resources²			0.027	-0.44	
Nectar resources		-0.059		-0.111	
Species number	0.207	0.247	0.198	0.233	0.206
Nectar res. × Sp. number				0.585	
AIC	135.8	137.5	137.7	137.8	137.8
ΔAIC from best model	0.00	1.76	1.89	1.97	1.98
Marginal R²	0.43	0.46	0.44	0.39	0.43
Conditional R²	0.44	0.47	0.45	0.40	0.44

Brassicogethes aeneus	**Models with ΔAIC < 2**						
**Explanatory fixed variables**	1	2	3	4	5	6	7
Intercept	-2.296	-2.283	-2.299	-2.268	-2.247	-2.266	-2.299
Functional dispersion²				-0.031	-0.042		
Functional dispersion			0.009			-0.001	
Nectar resources²	0.114	0.105	0.117	0.113	0.104	0.111	0.119
Nectar resources					-0.001		0.033
Species number²						-0.029	
Species number	0.113			0.109		0.122	0.115
AIC	152.7	153.0	153.7	154.4	154.5	154.6	154.7
ΔAIC from best model	0.00	0.22	1.02	1.72	1.85	1.88	1.9
Marginal R²	0.32	0.23	0.28	0.33	0.33	0.33	0.32
Conditional R²	0.35	0.26	0.31	0.36	0.36	0.35	0.35

Parasitism at 20 m from the strip

Ceutorhynchus pallidactylus	**Models with ΔAIC < 2**						
**Explanatory fixed variables**	1	2	3	4	5	6	7
Intercept	-1.335	-1.306	-1.332	-1.326	-1.330	-1.301	-1.325
Functional dispersion	-0.193		-0.249		-0.297		-0.363
Nectar resources			0.164			0.082	0.166
Species number				-0.121	0.138		0.135
AIC	65.5	65.8	65.9	66.9	67.1	67.4	67.4
ΔAIC from best model	0.00	0.36	0.40	1.46	1.65	1.94	1.96
Marginal R²	0.16	0	0.26	0.07	0.18	0.03	0.28
Conditional R²	0.17	0.00	0.27	0.07	0.19	0.03	0.29

Brassicogethes aeneus	**Models with ΔAIC < 2**									
**Explanatory fixed variables**	1	2	3	4	5	6	7	8	9	10
Intercept	-2.267	-2.293	-2.298	-2.345	-2.337	-2.376	-2.244	-2.218	-2.278	-2.266
Functional dispersion²			0.258	0.077						
Functional dispersion	0.247	0.302	0.354	0.350	0.452		0.441	0.242		0.262
Nectar resources²										
Nectar resources		0.039			0.036		0.026			0.029
Species number²								-0.049		
Species number	-0.302	-0.339	-0.290	-0.362	-0.412		-0.353	-0.288	-0.115	-0.309
Func. disp. × Nectar res.					0.202		0.331			
Func. disp. × Sp. number			-0.302				-0.186			
Nectar res. × Sp. number		0.184								
AIC	152.0	153.0	154.0	154.0	154.2	154.6	154.8	155.3	155.7	155.7
ΔAIC from best model	0.00	0.97	1.03	1.03	1.24	1.35	1.52	1.78	1.85	1.89
Marginal R²	0.36	0.24	0.28	0.37	0.36	0.31	0.32	0.36	0.36	0.36
Conditional R²	0.57	0.48	0.52	0.58	0.57	0.54	0.54	0.57	0.57	0.57

Table S8 (continued).

Parasitism at 5 m from the strip

Dasineura brassicae	Models with ΔAIC < 2							
Explanatory fixed	1	2	3	4	5	6	7	8
Intercept	-0.896	-0.899	-0.903	-0.909	-0.900	-0.913	-0.905	-0.901
Functional dispersion²			0.040	0.057			0.044	
Functional dispersion	0.091			0.055		0.081		
Nectar resources²					0.033	0.049		
Nectar resources	-0.067					-0.082		-0.035
Species number								0.039
Nectar res. × Sp. number								
AIC	197.5	197.6	197.7	198.0	198.2	198.2	198.6	198.7
ΔAIC from best model	0.00	0.02	0.14	0.49	0.63	0.63	1.10	1.15
Marginal R^2	0.21	0.00	0.07	0.13	0.05	0.19	0.11	0.03
Conditional R^2	0.41	0.25	0.30	0.35	0.29	0.39	0.33	0.27

Parasitism at 20 m from the strip

Dasineura brassicae	Models with ΔAIC < 2								
Explanatory fixed variables	1	2	3	4	5	6	7	8	9
Intercept	-0.936	-0.881	-0.902	-0.850	-0.902	-0.934	-0.957	-0.935	-0.936
Functional dispersion²		-0.059	-0.055	-0.090					
Functional dispersion			-0.096	-0.102		-0.049			
Nectar resources²					-0.036				
Nectar resources			-0.127						-0.012
Species number²			0.260				0.021		
Species number								-0.012	
Func. disp. × Nectar res.			0.437						
AIC	207.1	208.0	208.6	208.7	208.7	208.7	209.0	209.0	209.0
ΔAIC from best model	0.00	0.97	1.56	1.63	1.64	1.64	1.94	1.98	1.98
Marginal R^2	0.00	0.67	0.66	0.67	0.63	0.60	0.68	0.70	0.65
Conditional R^2	0.70	0.67	0.66	0.67	0.63	0.60	0.68	0.70	0.65

Multi-species parasitism	Models with ΔAIC < 2								
Explanatory fixed variables	1	2	3	4	5	6	7	8	9
Intercept	1.862	1.843	1.839	1.830	1.848	1.850	1.883	1.860	1.852
Threshold	-1.688	-1.681	-1.689	-1.681	-1.685	-1.684	-1.806	-1.689	-1.684
Functional dispersion²	0.055	0.073	0.020	0.027	0.068	0.067	0.087	0.038	0.064
Funct. disp.² × threshold	-0.271	-0.279	-0.270	-0.279	-0.274	-0.276	-0.444	-0.270	-0.275
Functional dispersion						0.028			-0.049
Nectar resources²				0.059				0.019	
Nectar resources					0.030				
Species number²			0.058				-0.052		
Sp. number² × threshold							0.284		
Species number		0.055		0.073					
AIC	782.5	782.9	783.6	783.9	784.2	784.2	784.2	784.4	784.4
ΔAIC from best model	0.00	0.34	1.08	1.40	1.63	1.66	1.67	1.88	1.90
Marginal R^2	0.50	0.51	0.50	0.51	0.50	0.50	0.51	0.50	0.51
Conditional R^2	0.50	0.51	0.51	0.51	0.50	0.51	0.51	0.50	0.51

Multi-species parasitism	Models with ΔAIC < 2								
Explanatory fixed variables	1	2	3	4	5	6	7	8	9
Intercept	1.852	1.995	1.901	1.999	1.966	1.900	1.953	2.003	1.900
Threshold	-1.419	-1.787	-1.787	-1.787	-1.787	-1.787	-1.787	-1.807	-1.787
Functional dispersion²				0.165			-0.053		
Functional dispersion						0.083			
Nectar resources²					0.053				
Nectar resources	-0.133	-0.134	-0.112	-0.123	-0.136	-0.176	-0.129	-0.070	-0.154
Nectar res. × threshold								-0.166	
Species number²	0.053	-0.098		-0.269	-0.121			-0.098	
Sp. number² × threshold	-0.394								
Species number									0.058
AIC	387.1	387.2	387.4	388.0	388.4	388.6	388.7	388.8	388.9
ΔAIC from best model	0.00	0.18	0.35	0.95	1.17	1.30	1.53	1.62	1.72
Marginal R^2	0.49	0.49	0.48	0.49	0.49	0.48	0.48	0.49	0.48
Conditional R^2	0.50	0.49	0.49	0.49	0.49	0.48	0.48	0.49	0.48

Supplementary Table S9 (full version of Table 1) Effects of the composition and structure (proportion of plant cover providing accessible nectar, species richness and functional dispersion) of the flower strip plant communities on the rates of parasitism in five herbivorous crop pests and on global multi-species parasitism (quantified via a multi-threshold approach). All possible combinations of the plant community variables (nectar resources, species richness and functional diversity, with both linear and quadratic effects) and their interactions were compared. The best models were ranked according to their AIC (Table S8) and we present the results for the conditional average of best models. Generalized linear mixed effect models were used, assuming a binomial (parasitism rates) or Poisson (multi-species parasitism) distribution, with strip as a random effect. All explanatory variables were scaled. We report the relative importance for each predictor (weight). “n” is the number of observations for each response variable.

Parasitism at 5 m from the strip

Bruchus rufimanus **(n=27)**	**Cond. averaged model**			
**Explanatory fixed variables**	**Effect ± SE**	**z-value**	**P (>z)**	**weight**
Intercept	-0.827 ± 0.038	20.249	**$< 10^{-4}$**	
Functional dispersion²	-0.071 ± 0.029	2.286	**0.02**	1
Functional dispersion	-0.038 ± 0.038	0.939	0.35	0.19
Nectar resources²	0.014 ± 0.026	0.501	0.62	0.13
Nectar resources	0.087 ± 0.032	2.610	**0.009**	1
Species number²	0.002 ± 0.032	0.064	0.95	0.12
Species number	-0.044 ± 0.036	1.149	0.25	0.24

Psylliodes chrysocephala **(n=27)**	**Cond. averaged model**			
**Explanatory fixed variables**	**Effect ± SE**	**z-value**	**P (>|z|)**	**weight**
Intercept	-2.409 ± 0.267	8.568	**$< 10^{-4}$**	
Functional dispersion²	-0.224 ± 0.178	1.204	0.23	0.33
Functional dispersion	-0.194 ± 0.202	0.907	0.36	0.13
Nectar resources²	0.218 ± 0.146	1.439	0.15	0.33
Nectar resources	0.382 ± 0.166	2.183	**0.02**	0.78
Species number²	-0.115 ± 0.219	0.497	0.62	0.10
Species number	0.044 ± 0.217	0.195	0.85	0.19

Ceutorhynchus pallidactylus **(n=27)**	**Cond. averaged model**			
**Explanatory fixed variables**	**Effect ± SE**	**z-value**	**P (>|z|)**	**weight**
Intercept	-1.256 ± 0.102	11.726	**$< 10^{-4}$**	
Functional dispersion²	-0.011 ± 0.076	0.136	0.89	0.11
Nectar resources²	-0.202 ± 0.291	0.681	0.50	0.23
Nectar resources	-0.083 ± 0.116	0.683	0.49	0.24
Species number	0.214 ± 0.108	1.878	0.06	1
Nectar res. × Sp. number	0.585 ± 0.271	2.027	**0.04**	0.11

Parasitism at 20 m from the strip

Bruchus rufimanus **(n=27)**	**Cond. averaged model**			
**Explanatory fixed variables**	**Effect ± SE**	**z-value**	**P (>|z|)**	**weight**
Intercept	-0.711 ± 0.070	9.890	**$< 10^{-4}$**	
Functional dispersion²	-0.108 ± 0.048	2.185	**0.02**	0.83
Functional dispersion	-0.078 ± 0.065	1.138	0.26	0.43
Nectar resources²	0.060 ± 0.039	1.465	0.14	0.60
Nectar resources	-0.059 ± 0.051	1.112	0.27	0.30
Species number²	0.023 ± 0.053	0.433	0.67	0.19
Species number	0.122 ± 0.070	1.674	0.09	0.61
Func. disp. × Sp. number	-0.072 ± 0.032	2.112	**0.03**	0.05

Psylliodes chrysocephala **(n=12)**	**Cond. averaged model**			
**Explanatory fixed variables**	**Effect ± SE**	**z-value**	**P (>|z|)**	**weight**
Intercept	-2.121 ± 0.305	6.149	**$< 10^{-4}$**	
Functional dispersion	-0.073 ± 0.202	0.317	0.75	0.17
Nectar resources	-0.253 ± 0.272	0.820	0.41	0.24
Species number	-0.084 ± 0.216	0.341	0.73	0.17

Ceutorhynchus pallidactylus **(n=13)**	**Cond. averaged model**			
**Explanatory fixed variables**	**Effect ± SE**	**z-value**	**P (>|z|)**	**weight**
Intercept	-1.323 ± 0.118	9.800	**$< 10^{-4}$**	
Functional dispersion	-0.252 ± 0.170	1.301	0.19	0.61
Nectar resources	0.145 ± 0.131	0.963	0.34	0.36
Species number	0.037 ± 0.218	0.154	0.88	0.30

Table S9 (continued).

***Brassicogethes aeneus* (n=27)**	**Cond. averaged model**			
**Explanatory fixed variables**	**Effect ± SE**	**z-value**	**P (>|z|)**	**weight**
Intercept	-2.284 ± 0.093	23.206	**$< 10^{-4}$**	
Functional dispersion^2	-0.036 ± 0.060	0.568	0.57	0.19
Functional dispersion	0.057 ± 0.108	0.504	0.61	0.22
Nectar resources^2	0.111 ± 0.045	2.367	**0.01**	1
Nectar resources	-0.001 ± 0.099	0.092	0.93	0.08
Species number^2	-0.029 ± 0.082	0.328	0.74	0.09
Species number	0.114 ± 0.079	1.351	0.18	0.58

***Dasineura brassicae* (n=25)**	**Cond. averaged model**			
**Explanatory fixed variables**	**Effect ± SE**	**z-value**	**P (>|z|)**	**weight**
Intercept	-0.894 ± 0.083	10.110	**$< 10^{-4}$**	
Functional dispersion^2	0.032 ± 0.055	0.554	0.58	0.29
Functional dispersion	0.080 ± 0.062	1.220	0.22	0.46
Nectar resources^2	0.037 ± 0.032	1.074	0.28	0.29
Nectar resources	-0.057 ± 0.049	1.098	0.27	0.52
Species number	0.019 ± 0.062	0.285	0.78	0.25
Nectar res. × Sp. number	0.087 ± 0.048	1.684	0.09	0.06

**Multispecies parasitism (n=243)**	**Cond. averaged model**			
**Explanatory fixed variables**	**Effect ± SE**	**z-value**	**P (>|z|)**	**weight**
Intercept	1.851 ± 0.090	20.40	**$< 10^{-4}$**	
Threshold	-1.695 ± 0.188	8.977	**$< 10^{-4}$**	1
Functional dispersion^2	0.055 ± 0.062	0.882	0.38	1
Functional disp.^{2} × threshold	-0.289 ± 0.036	2.114	**0.03**	1
Functional dispersion	-0.008 ± 0.072	0.116	0.91	0.16
Species number^2	0.011 ± 0.100	0.108	0.91	0.20
Species number^2 × threshold	0.284 ± 0.223	1.230	0.22	0.09
Species number	0.067 ± 0.051	1.306	0.19	0.34
Nectar resources^2	0.042 ± 0.062	0.671	0.50	0.18
Nectar resources	0.030 ± 0.049	0.606	0.54	0.09

***Brassicogethes aeneus* (n=27)**	**Cond. averaged model**			
**Explanatory fixed variables**	**Effect ± SE**	**z-value**	**P (>|z|)**	**weight**
Intercept	-2.282 ± 0.109	19.954	**$< 10^{-4}$**	
Functional dispersion^2	0.168 ± 0.148	1.090	0.28	0.19
Functional dispersion	0.315 ± 0.159	1.895	0.06	0.86
Nectar resources^2	-0.012 ± 0.061	0.191	0.85	0.06
Nectar resources	0.008 ± 0.091	0.082	0.93	0.42
Species number^2	-0.049 ± 0.105	0.442	0.66	0.07
Species number	-0.313 ± 0.143	2.085	**0.03**	0.92
Func. disp. × Nectar res.	0.263 ± 0.155	1.613	0.11	0.16
Func. disp. × Sp. number	-0.182 ± 0.209	0.837	0.40	0.23
Nectar res. × Sp. number	0.184 ± 0.107	1.613	0.11	0.10

***Dasineura brassicae* (n=26)**	**Cond. averaged model**			
**Explanatory fixed variables**	**Effect ± SE**	**z-value**	**P (>|z|)**	**weight**
Intercept	-0.915 ± 0.095	9.159	**$< 10^{-4}$**	
Functional dispersion^2	-0.218 ± 0.254	0.846	0.40	0.34
Functional dispersion	-0.082 ± 0.092	0.851	0.40	0.30
Nectar resources^2	0.036 ± 0.059	0.575	0.57	0.10
Nectar resources	-0.076 ± 0.109	0.664	0.51	0.18
Species number^2	0.152 ± 0.158	0.942	0.35	0.18
Species number	-0.012 ± 0.079	0.143	0.89	0.08
Func. disp. × Nectar res.	0.437 ± 0.194	2.107	**0.03**	0.10

**Multispecies parasitism (n=117)**	**Cond. averaged model**			
**Explanatory fixed variables**	**Effect ± SE**	**z-value**	**P**	**weight**
Intercept	1.940 ± 0.127	15.135	**$< 10^{-4}$**	
Threshold	-1.737 ± 0.269	6.580	**$< 10^{-4}$**	1
Functional dispersion^2	0.072 ± 0.159	0.449	0.65	0.16
Functional dispersion	0.067 ± 0.084	0.792	0.43	0.13
Nectar resources^2	0.052 ± 0.052	0.997	0.32	0.08
Nectar resources	-0.129 ± 0.075	1.708	0.09	1
Nectar ress. × threshold	-0.164 ± 0.211	0.768	0.44	0.12
Species number^2	-0.089 ± 0.139	0.634	0.53	0.62
Species number^2 × threshold	-0.394 ± 0.251	1.554	0.12	0.14
Species number	0.049 ± 0.075	0.643	0.52	0.12

Supplementary Table S10. Results of neutral models in which the cover of plants providing accessible nectar is replaced by the cover of randomly selected plant species (among all plants or among the only flowering plants). We show the AIC of the best models (same approach as in Table 1 and Table S9) averaged over 1000 iterations, the comparison with the AIC of the trait matching models (Δ AIC = AIC of neutral model – AIC of trait-matching model) and the proportion of neutral models with AIC lower than the AIC of the trait-matching models (P).

	**Parasitism at 5 m from the strip**			**Parasitism at 20 m from the strip**		
**Neutral model with all plant species**	**AIC (mean ± SD)**	**Δ AIC**	**P**	**AIC (mean ± SD)**	**Δ AIC**	**P**
Bruchus rufimanus	213.73 ± 3.50	14.73	0.006	237.315 ± 5.737	11.12	0.048
Psylliodes chrysocephala	91.956 ± 2.151	3.36	0.082	38.177 ± 1.521	-14.72	1
Ceutorhynchus pallidactylus	138.033 ± 782	2.23	0.181	65.737 ± 1.377	0.24	0.294
Brassicogethes aeneus	158.405 ± 2.380	5.71	0.042	152.295 ± 2.310	0.30	0.383
Dasineura brassicae	189.148 ± 4.669	37.15	0	239.807 ± 11.104	32.71	0.009
**Neutral model with only flowering plant species**						
Bruchus rufimanus	218.998 ± 4.245	19.99	0.002	237.193 ± 4.318	10.99	0.025
Psylliodes chrysocephala	89.544 ± 1.699	0.94	0.275	37.481 ± 1.770	-15.42	1
Ceutorhynchus pallidactylus	139.977 ± 0.836	4.18	0.006	65.838 ± 1.008	0.34	0.303
Brassicogethes aeneus	158.335 ± 2.750	5.64	0.059	152.322 ± 2.314	0.32	0.302
Dasineura brassicae	189.606 ± 4.7729	37.61	0	239.878 ± 10.748	32.78	0.006

Supplementary Table S11. Effects of the composition and structure of the flower strip plant communities on the rates of parasitism in five herbivorous crop pests and on global multi-species parasitism. In complement to the results of Table 1, we analysed separately the gradients of sown functional diversity and species richness. The effect of sown functional diversity was analysed on a subset of medium species richness assemblages (panel A). The effect of sown richness diversity was analysed on the subset of high functional diversity assemblages (panel B).

A – Only medium species richness assemblages (LFMS and HFMS)

Parasitism at 5 m from the strip

Bruchus rufimanus **(n=12)**	**Cond. averaged model**			
**Explanatory fixed variables**	**Effect ± SE**	**z-value**	**P (>z)**	**weight**
Intercept	-0.724 ± 0.080	7.808	**$< 10^{-4}$**	
Functional dispersion^2	-0.105 ± 0.052	1.635	0.102	0.83
Functional dispersion	-0.152 ± 0.057	2.210	**0.027**	1
Nectar resources^2	-0.052 ± 0.044	0.943	0.346	0.09
Nectar resources	0.103 ± 0.050	1.665	0.096	0.91
Species number^2	-0.052 ± 0.044	0.943	0.346	0.11
Species number	-0.046 ± 0.056	0.646	0.518	0.37
Func. disp. × Sp. number	-0.172 ± 0.158	0.764	0.445	0.08
Nectar res. × Sp. number	0.084 ± 0.049	1.273	0.203	0.20
Func. disp. × Nectar res.	-0.044 ± 0.082	0.449	0.654	0.16

Psylliodes chrysocephala **(n=12)**	**Cond. averaged model**			
**Explanatory fixed variables**	**Effect ± SE**	**z-value**	**P (>|z|)**	**weight**
Intercept	-2.540 ± 0.587	3.744	**$< 10^{-4}$**	
Functional dispersion^2	-0.050 ± 0.237	0.184	0.854	0.12
Functional dispersion	0.075 ± 0.225	0.28	0.774	0.11
Nectar resources^2	0.143 ± 0.170	0.741	0.459	0.24
Nectar resources	0.270 ± 0.240	0.961	0.337	0.44
Species number^2	-0.331 ± 0.374	0.754	0.451	0.15
Species number	0.195 ± 0.366	0.466	0.641	0.17

Ceutorhynchus pallidactylus **(n=12)**	**Cond. averaged model**			
**Explanatory fixed variables**	**Effect ± SE**	**z-value**	**P (>|z|)**	**weight**
Intercept	-1.090 ± 0.126	7.464	**$< 10^{-4}$**	
Functional dispersion^2	-0.010 ± 0.105	0.082	0.935	0.06
Functional dispersion	-0.088 ± .144	0.529	0.597	0.37
Nectar resources^2	-0.214 ± 0.318	0.626	0.531	0.15
Nectar resources	0.102 ± 0.173	0.495	0.620	0.39
Species number^2	0.008 ± 0.098	0.067	0.947	0.06
Species number	0.171 ± 0.167	0.866	0.386	0.37
Nectar res. × Sp. number	0.519 ± 0.365	1.250	0.211	0.15
Nectar res. × Func. dispersion	0.403 ± 0.219	1.466	0.143	0.20

Parasitism at 20 m from the strip

Bruchus rufimanus **(n=12)**	**Cond. averaged model**			
**Explanatory fixed variables**	**Effect ± SE**	**z-value**	**P (>|z|)**	**weight**
Intercept	-0.661 ± 0.069	5.601	**$< 10^{-4}$**	
Functional dispersion^2	-0.346 ± 0.084	2.455	**0.014**	1
Functional dispersion	-0.116 ± 0.050	1.360	0.174	1
Nectar resources^2	0.177 ± 0.051	2.074	0.038	1
Nectar resources	-0.165 ± 0.053	1.805	0.071	1
Species number^2	0.026 ± 0.053	0.307	0.759	0.33
Species number	-0.056 ± 0.061	0.519	0.605	0.69
Func. disp. × Sp. number	-0.119 ± 0.156	0.349	0.727	0.11
Nectar res. × Sp. number	-0.089 ± 0.053	0.902	0.367	0.55
Func. disp. × Nectar res.	0.373 ± 0.088	2.465	**0.014**	1

Psylliodes chrysocephala **(n=12)**	**Cond. averaged model**			
**Explanatory fixed variables**	**Effect ± SE**	**z-value**	**P (>|z|)**	**weight**
The model does not converge.				

Ceutorhynchus pallidactylus **(n=12)**	**Cond. averaged model**			
**Explanatory fixed variables**	**Effect ± SE**	**z-value**	**P (>|z|)**	**weight**
The model does not converge.				

Table S11 A (continued).

***Brassicogethes aeneus* (n=12)**	**Cond. averaged model**			
**Explanatory fixed variables**	**Effect ± SE**	**z-value**	**P (>|z|)**	**weight**
Intercept	-2.348 ± 0.164	12.724	**$< 10^{-4}$**	
Functional dispersion^2	0.156 ± 0.147	0.894	0.371	0.22
Functional dispersion	-0.081 ± 0.128	0.522	0.602	0.06
Nectar resources^2	0.069 ± 0.057	1.041	0.298	0.57
Nectar resources	0.025 ± 0.175	0.128	0.898	0.29
Species number^2	0.042 ± 0.109	0.320	0.749	0.05
Species number	0.191 ± 0.114	1.422	0.155	0.75

***Brassicogethes aeneus* (n=12)**	**Cond. averaged model**			
**Explanatory fixed variables**	**Effect ± SE**	**z-value**	**P (>|z|)**	**weight**
Intercept	-2.364 ± 0.168	11.853	**$< 10^{-4}$**	
Functional dispersion^2	0.045 ± 0.149	0.244	0.807	0.11
Functional dispersion	0.265 ± 0.285	0.861	0.389	1
Nectar resources^2	0.008 ± 0.041	0.164	0.870	0.11
Nectar resources	0.322 ± 0.277	1.003	0.316	0.37
Species number^2	0.215 ± 0.107	1.639	0.101	0.74
Species number	-0.215 ± 0.251	0.793	0.428	1
Func. disp. × Nectar res.	-1.065 ± 0.455	1.824	0.068	0.26
Func. disp. × Sp. number	0.020 ± 0.114	0.141	0.888	0.11
Nectar res. × Sp. number	0.600 ± 0.229	1.997	0.058	0.26

***Dasineura brassicae* (n=12)**	**Cond. averaged model**			
**Explanatory fixed variables**	**Effect ± SE**	**z-value**	**P (>|z|)**	**weight**
Intercept	-1.080 ± 0.183	4.738	**$< 10^{-4}$**	
Functional dispersion^2	1.158 ± 0.108	1.000	0.318	0.63
Functional dispersion	0.193 ± 0.091	1.571	0.116	1
Nectar resources^2	0.008 ± 0.058	0.084	0.933	0.11
Nectar resources	-0.264 ± 0.104	1.764	0.078	0.78
Species number^2	0.211 ± 0.097	1.506	0.132	0.78
Species number	-0.022 ± 0.078	0.172	0.864	0.11
Nectar res. × Func. disp.	0.355 ± 0.147	1.670	0.095	0.78

***Dasineura brassicae* (n=26)**	**Cond. averaged model**			
**Explanatory fixed variables**	**Effect ± SE**	**z-value**	**P (>|z|)**	**weight**
Intercept	-0.807 ± 0.097	5.917	**$< 10^{-4}$**	
Functional dispersion^2	-0.154 ± 0.099	1.037	0.300	0.65
Functional dispersion	-0.189 ± 0.090	0.004	0.999	0.17
Nectar resources^2	0.036 ± 0.059	2.276	**0.023**	1
Nectar resources	0.100 ± 0.081	0.892	0.372	1
Species number^2	0.347 ± 0.119	2.137	**0.033**	1
Species number	-0.010 ± 0.059	0.116	0.908	1
Nectar res. × Sp. number	0.654 ± 0.186	2.179	**0.029**	1

**Multispecies parasitism (n=108)**	**Cond. averaged model**			
**Explanatory fixed variables**	**Effect ± SE**	**z-value**	**P (>|z|)**	**weight**
Intercept	1.754 ± 0.139	12.475	**$< 10^{-4}$**	
Threshold	-1.583 ± 0.224	7.000	**$< 10^{-4}$**	1
Functional dispersion^2	0.124 ± 0.082	1.497	0.134	0.75
Functional disp.^{2} × threshold	0.056 ± 0.263	0.211	0.833	0.05
Functional dispersion	-0.060 ± 0.056	0.071	0.284	0.29
Species number^2	0.030 ± 0.050	0.593	0.553	0.06
Species number × threshold	0.271 ± 0.217	1.236	0.217	0.14
Species number	0.030 ± 0.092	0.321	0.748	0.38
Nectar resources^2	-0.018 ± 0.037	0.478	0.633	0.05
Nectar resources	0.012 ± 0.056	0.211	0.833	0.05

**Multispecies parasitism (n=27)**	**Cond. averaged model**			
**Explanatory fixed variables**	**Effect ± SE**	**z-value**	**P**	**weight**
Intercept	1.821 ± 0.189	9.116	**$< 10^{-4}$**	
Threshold	-1.168 ± 0.313	3.525	**0.0004**	1
Functional dispersion^2	0.017 ± 0.068	0.238	0.812	0.13
Functional dispersion	-0.005 ± 0.036	0.137	0.891	0.11

Table S11 B – Only high functional diversity assemblages (HFLS, HFMS and HFHS)

Parasitism at 5 m from the strip

Bruchus rufimanus **(n=18)**	**Cond. averaged model**			
**Explanatory fixed variables**	**Effect ± SE**	**z-value**	**P (>z)**	**weight**
Intercept	-0.886 ± 0.038	21.397	**$< 10^{-4}$**	
Functional dispersion^2	-0.009 ± 0.036	0.242	0.809	0.100
Functional dispersion	-0.010 ± 0.031	0.294	0.769	0.101
Nectar resources^2	-0.027 ± 0.0637	0.675	0.500	0.126
Nectar resources	0.026 ± 0.033	0.739	0.460	0.133
Species number^2	-0.027 ± 0.028	0.946	0.344	0.164
Species number	-0.018 ± 0.031	0.539	0.590	0.114
Psylliodes chrysocephala **(n=18)**	**Cond. averaged model**			
**Explanatory fixed variables**	**Effect ± SE**	**z-value**	**P (>$\lvert z \rvert$)**	**weight**
Intercept	-1.892 ± 0.225	7.644	**$< 10^{-4}$**	
Functional dispersion^2	-0.560 ± 0.276	1.847	0.065	1
Functional dispersion	0.099 ± 0.237	0.379	0.705	0.16
Nectar resources^2	0.025 ± 0.150	0.151	0.880	0.15
Nectar resources	0.469 ± 0.193	2.211	**0.027**	1
Species number^2	0.008 ± 0.230	0.030	0.976	0.15
Species number	0.039 ± 0.177	0.200	0.842	0.15
Ceutorhynchus pallidactylus **(n=18)**	**Cond. averaged model**			
**Explanatory fixed variables**	**Effect ± SE**	**z-value**	**P (>$\lvert z \rvert$)**	**weight**
Intercept	-1.198 ± 0.146	7.586	**$< 10^{-4}$**	
Functional dispersion^2	-0.313 ± 0.202	1.432	0.152	0.45
Functional dispersion	0.188 ± 0.138	1.227	0.220	0.54
Nectar resources^2	-0.039 ± 0.273	0.135	0.892	0.25
Nectar resources	-0.028 ± 0.153	0.165	0.869	0.74
Species number^2	-0.229 ± 0.161	1.282	0.200	0.32
Species number	0.130 ± 0.209	0.570	0.569	0.67
Nectar res. × Sp. number	0.423 ± 0.260	1.522	0.128	0.38
Nectar res. × Func. disp.	0.439 ± 0.178	2.188	**0.029**	0.36
Func. disp × Sp. number	-0.498 ± 0.202	2.167	**0.030**	0.09

Parasitism at 20 m from the strip

Bruchus rufimanus **(n=18)**	**Cond. averaged model**			
**Explanatory fixed variables**	**Effect ± SE**	**z-value**	**P (>$\lvert z \rvert$)**	**weight**
Intercept	-0.836 ± 0.048	15.664	**$< 10^{-4}$**	
Functional dispersion^2	-0.005 ± 0.020	0.214	0.831	0.20
Functional dispersion	-0.082 ± 0.039	1.918	0.055	1
Nectar resources^2	0.107 ± 0.038	2.565	**0.010**	1
Nectar resources	-0.007 ± 0.024	0.255	0.798	0.22
Species number^2	-0.020 ± 0.031	0.619	0.536	0.45
Species number	0.152 ± 0.049	2.911	**0.004**	1
Func. disp. × Sp. number	-0.004 ± 0.015	0.241	0.809	0.13
Psylliodes chrysocephala **(n=12)**	**Cond. averaged model**			
**Explanatory fixed variables**	**Effect ± SE**	**z-value**	**P (>$\lvert z \rvert$)**	**weight**
The model does not converge.				
Ceutorhynchus pallidactylus **(n=13)**	**Cond. averaged model**			
**Explanatory fixed variables**	**Effect ± SE**	**z-value**	**P (>$\lvert z \rvert$)**	**weight**
The model does not converge.				

Table S11 B (continued).

Brassicogethes aeneus (n=18)	Cond. averaged model			
**Explanatory fixed variables**	**Effect ± SE**	**z-value**	**P (>|z|)**	**weight**
Intercept	-2.280 ± 0.095	22.102	**< 10^{-4}**	
Functional dispersion²	0.028 ± 0.059	0.433	0.655	0.12
Functional dispersion	0.034 ± 0.081	0.377	0.706	0.12
Nectar resources²	0.125 ± 0.047	2.418	**0.015**	1
Nectar resources	0.020 ± 0.106	0.175	0.861	0.11
Species number²	0.039 ± 0.087	0.413	0.680	0.12
Species number	0.095 ± 0.078	1.118	0.263	0.23

Brassicogethes aeneus (n=18)	Cond. averaged model			
**Explanatory fixed variables**	**Effect ± SE**	**z-value**	**P (>|z|)**	**weight**
Intercept	-2.298 ± 0.220	9.518	**< 10^{-4}**	
Functional dispersion²	0.308 ± 0.143	0.741	0.459	0.24
Functional dispersion	0.315 ± 0.159	1.967	**0.049**	0.94
Nectar resources²	-0.081 ± 0.096	0.766	0.444	0.32
Nectar resources	0.161 ± 0.133	1.080	0.280	0.40
Species number²	-0.032 ± 0.135	0.209	0.834	0.05
Species number	-0.415 ± 0.142	2.689	**0.007**	1
Func. disp. × Nectar res.	0.280 ± 0.159	1.574	0.115	0.40
Func. disp. × Sp. number	0.064 ± 0.174	0.343	0.732	0.19
Nectar res. × Sp. number	-0.024 ± 0.141	0.151	0.880	0.05

Dasineura brassicae (n=18)	Cond. averaged model			
**Explanatory fixed variables**	**Effect ± SE**	**z-value**	**P (>|z|)**	**weight**
Intercept	-0.911 ± 0.111	7.499	**< 10^{-4}**	
Functional dispersion²	0.062 ± 0.042	1.357	0.175	0.39
Functional dispersion	0.052 ± 0.052	0.907	0.364	0.33
Nectar resources²	-0.049 ± 0.072	0.647	0.517	0.14
Nectar resources	0.034 ± 0.056	0.542	0.588	0.29
Species number²	0.025 ±0.068	0.335	0.738	0.06
Species number	0.026 ± 0.047	0.507	0.612	0.12
Nectar res. × Func. disp.	0.123 ± 0.065	1.756	0.079	0.15

Dasineura brassicae (n=18)	Cond. averaged model			
**Explanatory fixed variables**	**Effect ± SE**	**z-value**	**P (>|z|)**	**weight**
Intercept	-0.661 ± 0.114	5.002	**< 10^{-4}**	
Functional dispersion²	-0.671 ± 0.175	3.304	**0.001**	1
Functional dispersion	-0.211 ± 0.106	1.728	0.084	1
Nectar resources²	-0.231 ± 0.079	2.536	0.011	1
Nectar resources	0.067 ± 0.077	0.748	0.454	1
Species number²	0.034 ± 0.086	0.341	0.732	0.21
Species number	0.130 ± 0.084	1.328	0.184	1
Func. disp. × Nectar res.	0.714 ± 0.183	3.391	**0.001**	1
Func. disp. × Sp. number	0.376 ± 0.151	2.148	0.317	0.21
Nectar res. × Sp. number	-0.130 ± 0.153	0.719	0.472	0.27

**Multispecies parasitism (n=162)**	Cond. averaged model			
**Explanatory fixed variables**	**Effect ± SE**	**z-value**	**P (>|z|)**	**weight**
Intercept	1.932 ± 0.087	22.005	**< 10^{-4}**	
Threshold	-1.936 ± 0.184	10.442	**< 10^{-4}**	1
Functional dispersion²	-0.016 ± 0.053	0.309	0.757	0.11
Functional dispersion	0.005 ± 0.046	0.103	0.918	0.10
Species number²	0.020 ± 0.052	0.380	0.704	0.11
Species number	0.050 ± 0.046	1.078	0.281	0.18
Nectar resources²	-0.001 ± 0.024	0.016	0.987	0.10
Nectar resources	0.022 ± 0.046	0.472	0.637	0.11

**Multispecies parasitism (n=63)**	Cond. averaged model			
**Explanatory fixed variables**	**Effect ± SE**	**z-value**	**P**	**weight**
Intercept	2.028 ± 0.195	10.189	**< 10^{-4}**	
Threshold	-2.651 ± 0.456	5.696	**< 10^{-4}**	1
Functional dispersion²	-0.026 ± 0.152	0.171	0.864	0.11
Functional dispersion	-0.031 ± 0.079	0.387	0.699	0.11
Nectar resources²	-0.102 ± 0.104	0.963	0.336	1
Nectar resources	0.008 ± 0.076	0.107	0.915	0.10
Nectar ress^{2}. × threshold	0.504 ± 0.219	2.255	**0.024**	1
Species number²	-0.056 ± 0.186	0.300	0.765	0.29
Species number² × threshold	-0.539 ± 0.480	1.097	0.273	0.11
Species number	0.003 ± 0.083	0.038	0.969	0.10

Supplementary figures

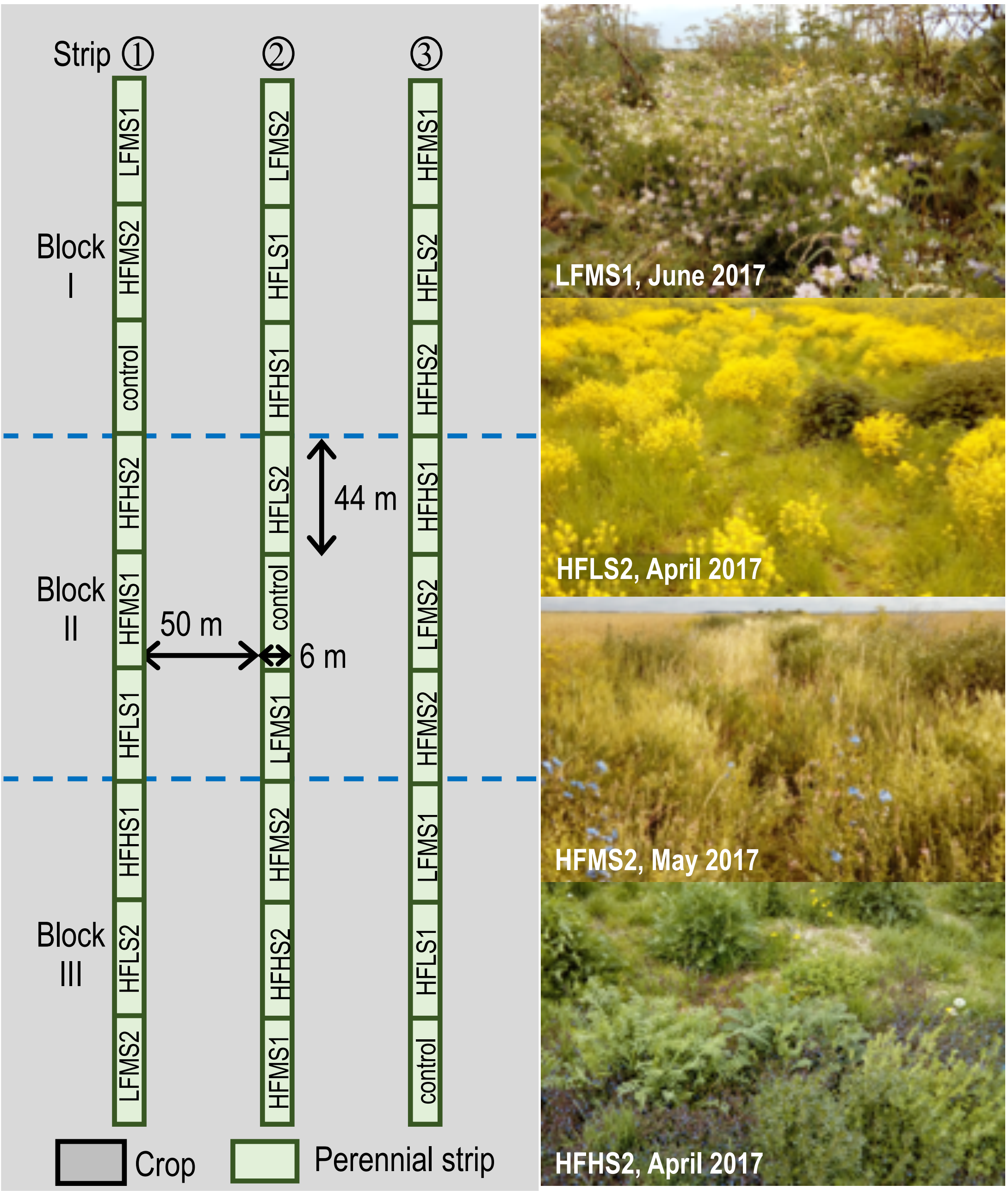

Supplementary Figure S2. Design of the experimental field (left) and illustrations of the different assemblages (right – Photo credits: A. Gardarin). The eight plant assemblages had a low or high functional diversity (LF or HF), a low, medium or high species richness (LS, MS or HS) and were composed of species from two different lists (1 or 2). The control treatment was sown with the crop species and managed similarly to the crop. This figure was created using Microsoft Powerpoint version 16.16.14 (https://www.microsoft.com).

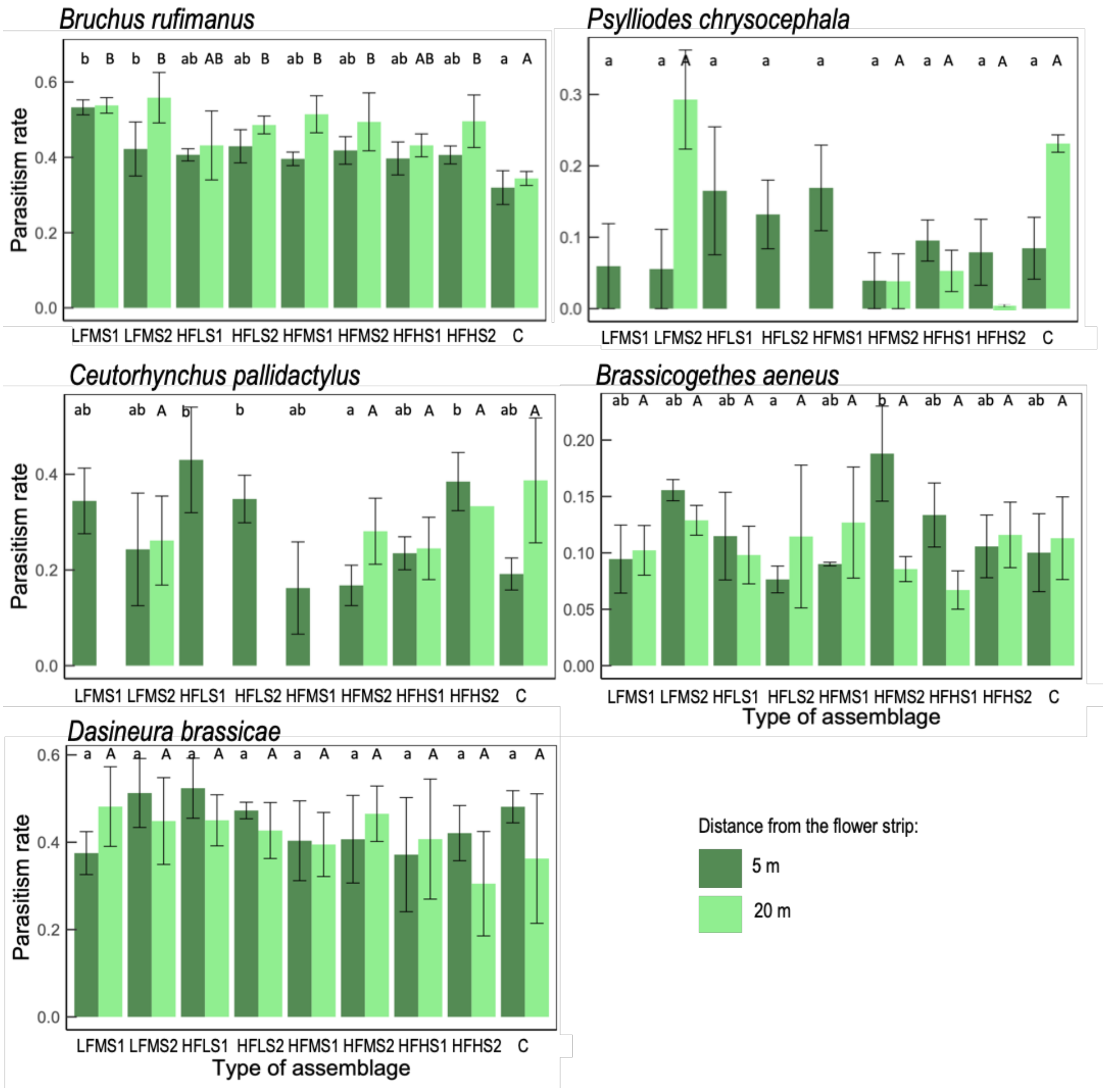

Supplementary Figure S3. Effect of the plant assemblages making up the flower strips on parasitism rates in five herbivorous insect pests in the adjacent crop. The eight plant assemblages have a low or high functional diversity (LF or HF, respectively), a low, medium or high species richness (LS, MS or HS respectively) and are composed of species from two different lists (1 or 2), and are compared with a control plot (C), on which the strip was sown with the same crop species as the field and managed in a similar manner. The different letters indicate significant differences between groups (*P*<0.05) between plant assemblages, within each category of distance (5 and 20 m from the strip). Not all treatments were studied at the 20 m distance. This figure was made using R version 3.6.3 (https://www.R-project.org/).

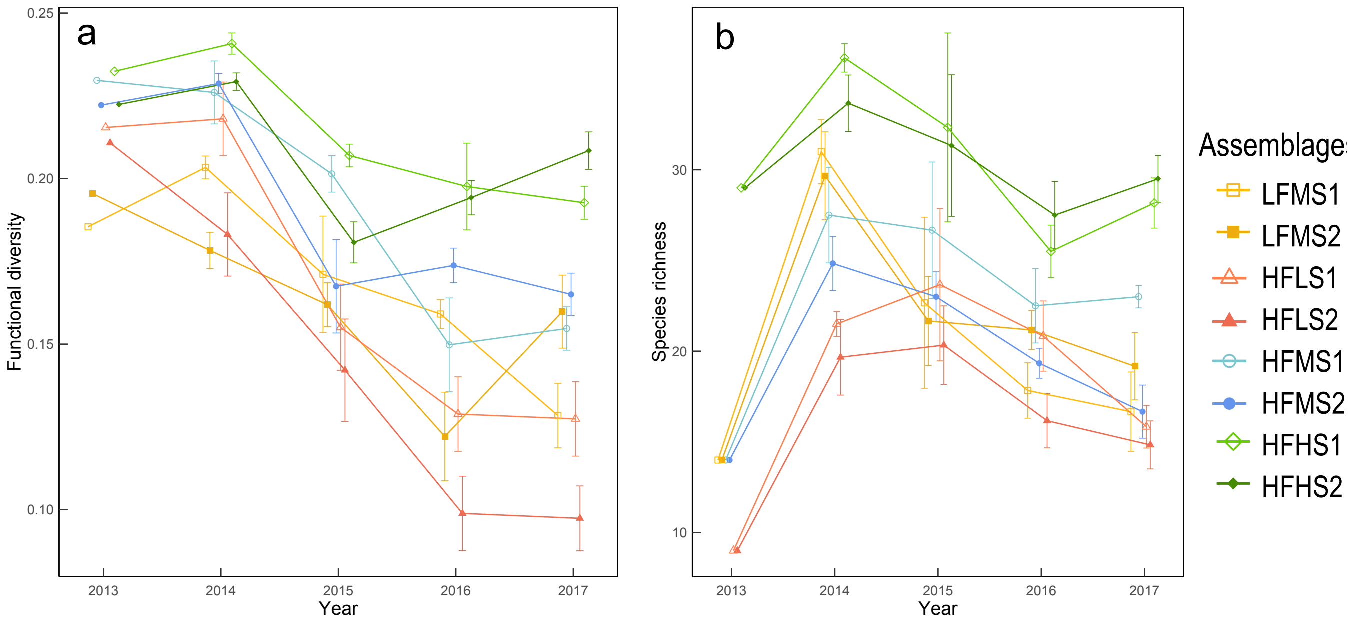

Supplementary Figure S4. Dynamics of the functional diversity (a) and species richness (b) of the eight sown plant assemblages (means ± standard errors). The data for 2013 are based on the seed mixtures sown, and the data for the next four years were obtained from field observations. The eight plant assemblages have a low or high functional diversity (LF or HF), a low, medium or high species richness (LS, MS or HS) and are composed of species from two different lists (1 or 2). This figure was made using R version 3.6.3 (https://www.R-project.org/).

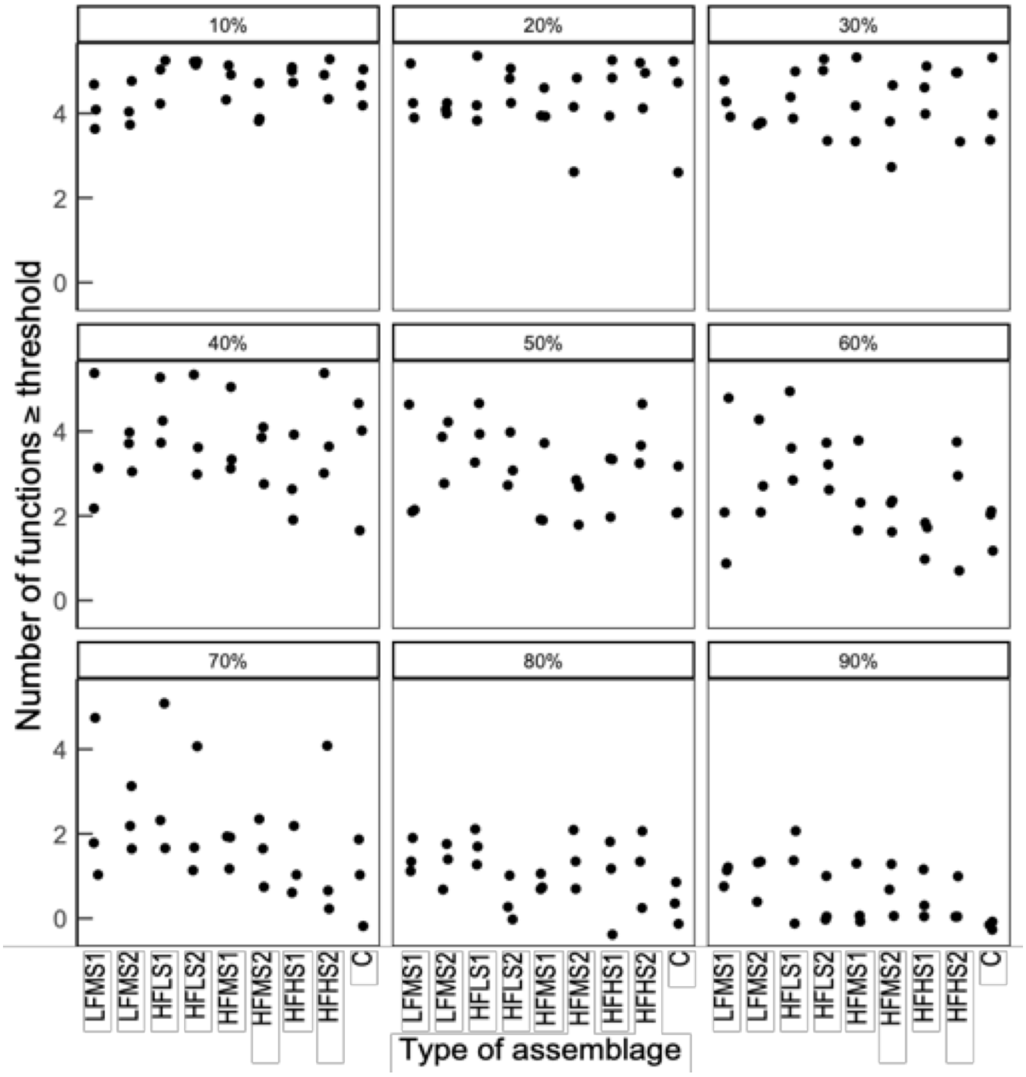

Supplementary Figure S5. Effect of the type of plant assemblage on multi-species parasitism in five herbivorous crop pests, measured at a distance of 5 m from the flower strips. Multi-species parasitism, quantified with a multi-threshold approach, was assessed as the number of herbivorous insect species for which the parasitism rate was above a given percentage (thresholds from 10 to 90%) of the maximum parasitism rate achieved for each species. This figure was made using R version 3.6.3 (https://www.R-project.org/).

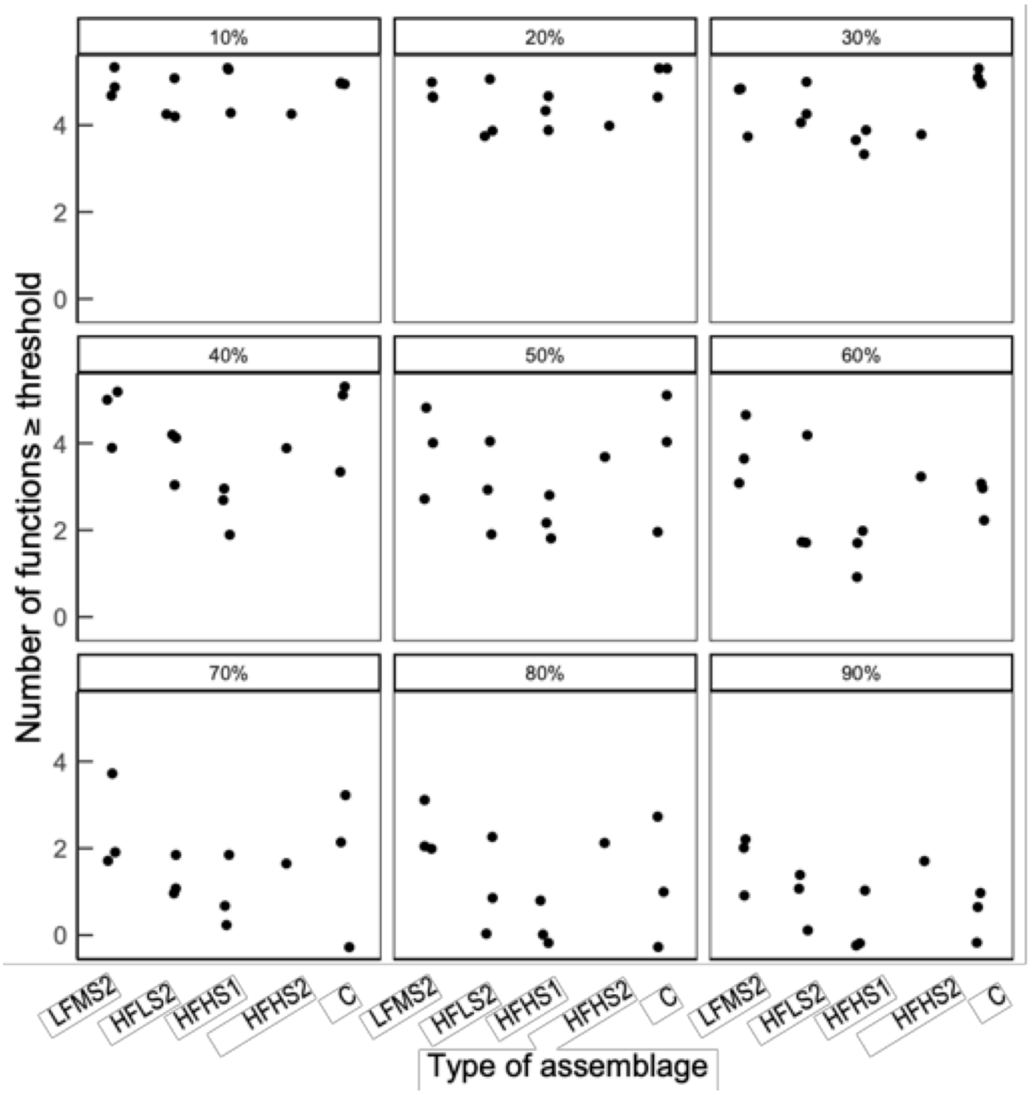

Supplementary Figure S6. Effect of the type of plant assemblage on multi-species parasitism in five herbivorous crop pests, measured at a distance of 20 m from the flower strips. Multi-species parasitism, quantified with a multi-threshold approach, was assessed as the number of herbivorous insect species for which the parasitism rate was above a given percentage (thresholds from 10 to 90%) of the maximum parasitism rate achieved for each species. This figure was made using R version 3.6.3 (https://www.R-project.org/).
